# Discovery of 4-(5-Membered)Heteroarylether-6-methylpicolinamide Negative Allosteric Modulators of Metabotropic Glutamate Receptor Subtype 5

**DOI:** 10.1021/acsmedchemlett.4c00481

**Published:** 2024-11-18

**Authors:** Elizabeth S. Childress, Rory A. Capstick, Katherine E. Crocker, Miranda L. Ledyard, Aaron M. Bender, Mallory A. Maurer, Natasha B. Billard, Hyekyung P. Cho, Alice L. Rodriguez, Colleen M. Niswender, Weimin Peng, Jerri M. Rook, Sichen Chang, Anna L. Blobaum, Olivier Boutaud, Analisa Thompson Gray, Carrie K. Jones, P. Jeffrey Conn, Andrew S. Felts, Craig W. Lindsley, Kayla J. Temple

**Affiliations:** † Warren Center for Neuroscience Drug Discovery, Vanderbilt University, Nashville, Tennessee 37232, United States; ‡ Department of Pharmacology, Vanderbilt University School of Medicine, Nashville, Tennessee 37232, United States; § Department of Chemistry, Vanderbilt University, Nashville, Tennessee 37232, United States; ∥ Department of Biochemistry, Vanderbilt University, Nashville, Tennessee 37232, United States; ⊥ Vanderbilt Kennedy Center, Vanderbilt University School of Medicine, Nashville, Tennessee 37232, United States; # Vanderbilt Brain Institute, Vanderbilt University School of Medicine, Nashville, Tennessee 37232, United States

**Keywords:** Metabotropic Glutamate Receptor Subtype 5, mGlu_5_, Negative Allosteric Modulator (NAM), Structure−Activity Relationship (SAR), Levodopa-Induced Dyskinesia, Alzheimer’s Disease, Pain, VU6043653

## Abstract

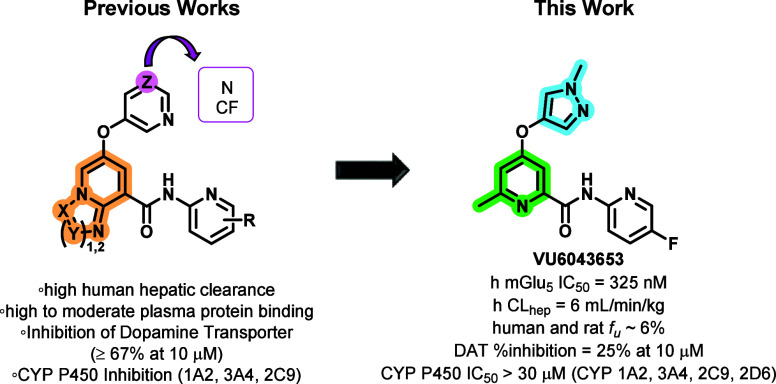

This Letter details our efforts to develop novel, non-acetylene-containing metabotropic glutamate receptor subtype 5 (mGlu_5_) negative allosteric modulators (NAMs) with improved pharmacological properties. This endeavor involved replacing the ether-linked pyrimidine moiety, a metabolic liability, with various 5-membered heterocycles. From this exercise, we identified **VU6043653**, a highly brain penetrant and selective mGlu_5_ NAM which displayed moderate potency against both human and rat mGlu_5_. Moreover, **VU6043653** has overall improved pharmacological and drug metabolism and pharmacokinetic profiles when compared to its predecessor compounds. Most notably, **VU6043653** exhibits low predicted human hepatic clearance, a clean cytochrome P450 profile, and minimal inhibition of the dopamine transporter.

The metabotropic glutamate (mGlu) receptors comprise a family of eight G protein-coupled receptors (GPCRs) that are activated by l-glutamic acid, the major excitatory neurotransmitter of the mammalian central nervous system (CNS). Once activated, the mGlu receptors modulate the strength of synaptic transmission. The eight mGlu receptors are divided into three groups based on structure and sequence homology, downstream signaling partners/pathways, as well as pharmacology. The mGlu_5_ receptor is widely expressed throughout the CNS and, alongside mGlu_1_, belongs to group I mGlu receptors, which are predominantly found postsynaptically and couple via G_q_ to the activation of phospholipase C (PLC).^1,2^ While designing selective orthostatic ligands that preferentially target one mGlu receptor over another has proven to be extremely challenging, one successful approach to selectively target individual mGlu receptor subtypes is via allosteric modulation. Negative allosteric modulators (NAMs) of mGlu_5_ are among the most advanced and widely investigated within the field of mGlu receptor allostery.^3−8^ Preclinical and clinical efficacy has established a multitude of potential therapeutic applications for small molecule mGlu_5_ NAMs, such as anxiety,^9,10^ Alzheimer’s disease,^11^ fragile X syndrome,^12−14^ autism spectrum disorder,^15,16^ levodopa-induced dyskinesia experienced by many Parkinson’s disease patients,^17−19^ gastroesophageal reflux disease,^20^ addiction disorder,^21−23^ major depressive disorder,^24−26^ obsessive-compulsive disorder,^27^ migraine, and pain.^28−31^ Early mGlu_5_ NAMs (e.g., **1** and **2**) were based on a key aryl/heterobiaryl acetylene pharmacophore, and this moiety has been carried throughout several subsequent medicinal chemistry optimization efforts (highlighted in Figure 1); however, alkynes, particularly those conjugated to an α-heteroatom, are potentially reactive functional groups.^32,33^ In fact, acetylene-based mGlu_5_ NAMs have been linked to hepatotoxicity and glutathione conjugation, as observed in both preclinical and clinical studies.^34^ AZD9272 (**7**) utilized an acetylene bioisostere, while fenobam (**3**) completely lacked the acetylene moiety. Both were advanced to clinical studies; however, their development was halted due to psychosis-like symptoms. Most importantly, further investigation into fenobam and AZD9272 attributed these symptoms to monoamine oxidase-B (MAO-B)-mediated mechanisms rather than mGlu_5_-mediated mechanisms.^35^ To date, no mGlu_5_ NAM has advanced to the market due, in part, to dose-limiting adverse events (such as hallucinations or psychotomimetic effects) observed in some clinical trials.^36^ Currently, TMP-301 (**9**) is the only clinical mGlu_5_ NAM devoid of the acetylene moiety and is undergoing Phase I clinical trials for substance abuse disorders.^37^ Therefore, endeavors in the field have shifted to identifying novel, non-acetylene-containing mGlu_5_ NAMs to avoid the pharmacophore-mediated adverse liabilities while exploiting the broad therapeutic utility of a selective mGlu_5_ NAM.

**Figure 1:**
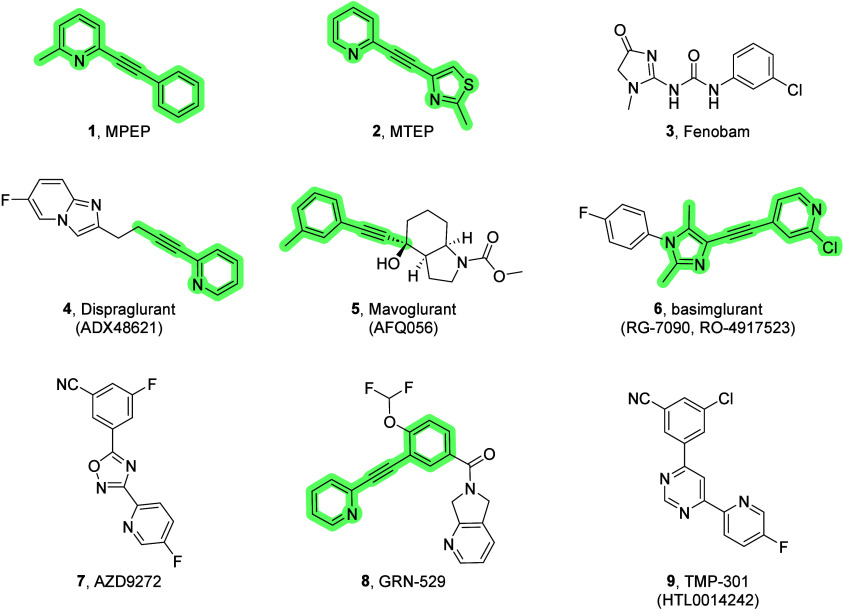
Prototypical mGlu_5_ NAM chemotypes. NAMs **1** and **2** were crucial early tool compounds, and NAMs **4**–**9** entered human clinical testing.

A major focus of our group has been the development of small molecule mGlu_5_ NAMs, which ultimately resulted in the identification of clinical candidate **10** (auglurant, **VU0424238**) (Figure 2).^38^ Unfortunately, **10** failed in development due to species-specific toxicities observed during a 28-day toxicologic assessment in cynomolgus monkeys, which were not previously observed in rats. Accumulation of a cyno-unique aldehyde oxidase (AO) metabolite was observed after 14 days and resulted in pronounced anemia (non-mechanism-based). Metabolism studies revealed the oxidation of the pyrimidine ring to a 6-oxopyrimidine metabolite, followed by the subsequent formation of a 2,6-oxopyrimidine metabolite. In humans, monkeys, and rats, it was determined that the formation of the 6-oxopyrimide metabolite was mediated by AO; however, there were apparent species differences between monkeys and rats in the enzyme involved in the formation of the 2,6-oxopyrimidine metabolite. While the second metabolite was mediated by AO metabolism in monkeys, it was determined that this process was mediated by xanthine oxidase (XO) metabolism rats.^39,40^ Therefore, it is possible that species differences in the involvement of AO/XO metabolism may play a role in the observed monkey-specific toxicity.

**Figure 2:**
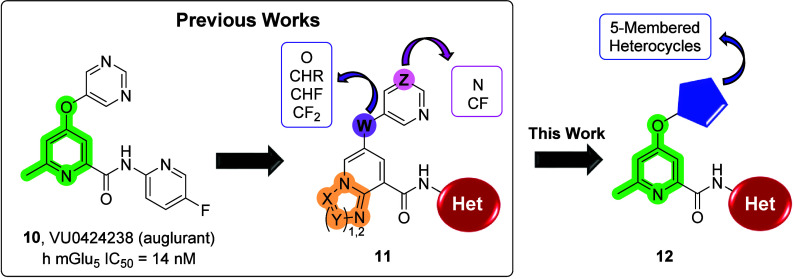
Previously published compounds that emerged from optimization of high-throughput screening hits: clinical candidate **VU0424238** (auglurant, **10**) and backup scaffold **11**. Further optimization led to potent mGlu_5_ NAMs **12**.

Attention was shifted to the development of backup analogs **11** in an effort to identify a compound devoid of AO metabolism. While this strategy allowed us to mitigate the role of AO, it did not allow us to fully eliminate this route of metabolism. Additionally, analogs **11** typically suffered from high predicted human hepatic clearance, high plasma protein binding, inhibition of cytochrome P450s (CYPs; in particular 1A2 but also 3A4 and 2C9), and/or inhibition of dopamine transporters (DAT). Thus, further optimization was required. This Letter describes the structure–activity relationship (SAR) development of novel mGlu_5_ NAMs (**12**) with various 5-membered heteroaryl groups as replacements for the pyrimidine moiety responsible for the AO-mediated metabolism observed in **10**.

The synthesis of analogs **22** was straightforward and began by reacting commercially available nitrile **13** with various commercially available 5-membered heteroaryl alcohols under basic conditions to afford the S_N_Ar products **14** (Scheme 1). Basic hydrolysis of nitriles **14** to the carboxylic acids **18** proceeded smoothly in 32–98% yield. Finally, conversion to the acid chloride and reaction with various heterocyclic amines *in situ* afforded analogs **22**. We next turned our attention to exploring further modifications to the central pyridine core with the synthesis of intermediates **15**–**17**. To prepare intermediate **15**, we utilized standard S_N_Ar protocols to react commercially available bromide **26** with alcohol **27** to provide intermediate **28**, which could then undergo a palladium-catalyzed cross-coupling with zinc cyanide to afford nitrile **15** (Scheme 2). Similar to intermediate **14**, nitrile **15** underwent basic hydrolysis to yield carboxylic acid **19**. Subsequent conversion to the acid chloride and reaction with various heterocyclic amines *in situ* afforded analogs **23**. The heterocyclic amines (R_4_) highlighted in Table 1 were select for evaluation based on prior endeavors in which these amines provided potent compounds with promising plasma protein binding and plasma clearance profiles.^38^

**Scheme 1:**
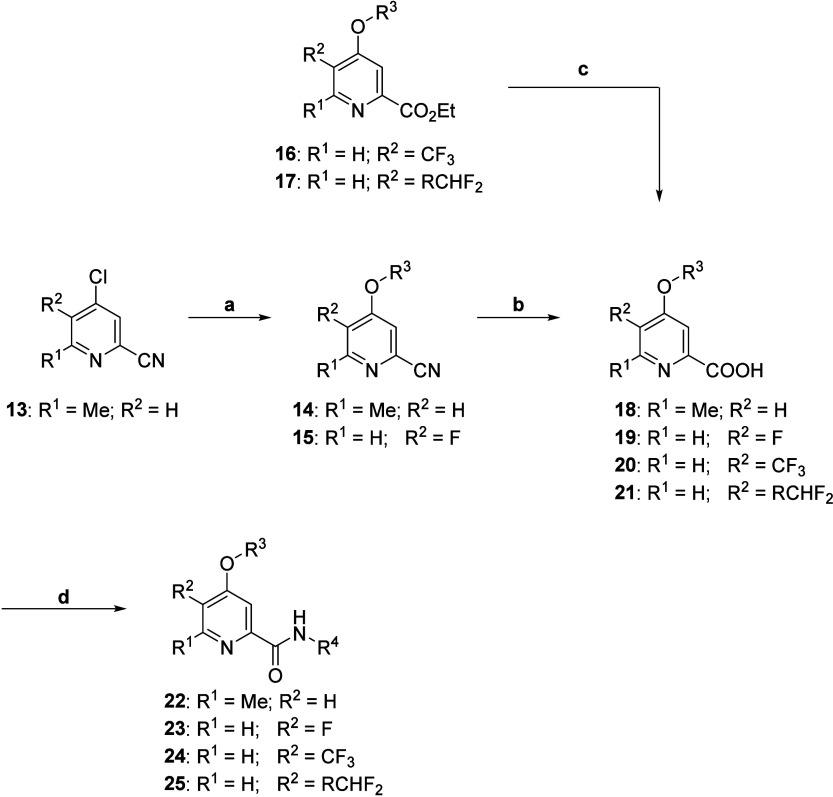
Synthesis of mGlu_5_ NAM Analogs **18–25** Reagents and conditions: (a) R^3^ = OH, K_2_CO_3_, DMF, μW 150 °C, 74–98%; (b) NaOH, EtOH/H_2_O, 100 °C, 32–98%; (c) NaOH, 1,4-dioxane/H_2_O, 98%; (d) POCl_3_, R^4^ = NH_2_, pyridine, 0 °C to r.t., 8–89%.

**Scheme 2:**
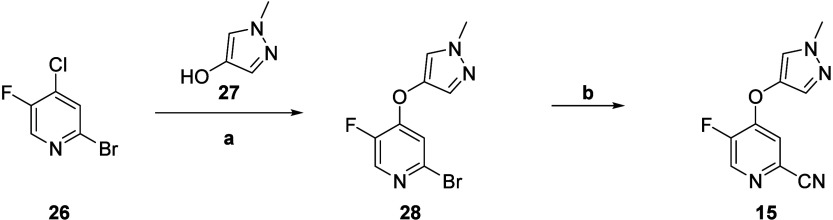
Synthesis of mGlu_5_ NAM Intermediate **15** Reagents and conditions: (a) Cs_2_CO_3_, DMSO, 79%; (b) Zn(CN)_2_, Pd(PPh_3_)_4_, DMF, μW 140 °C, 68%.

**Table 1:**
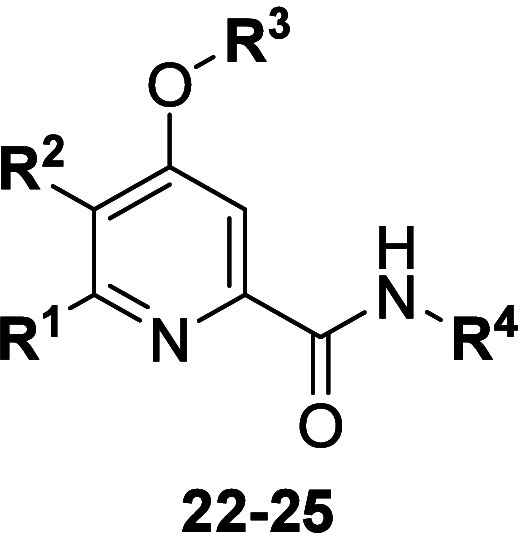

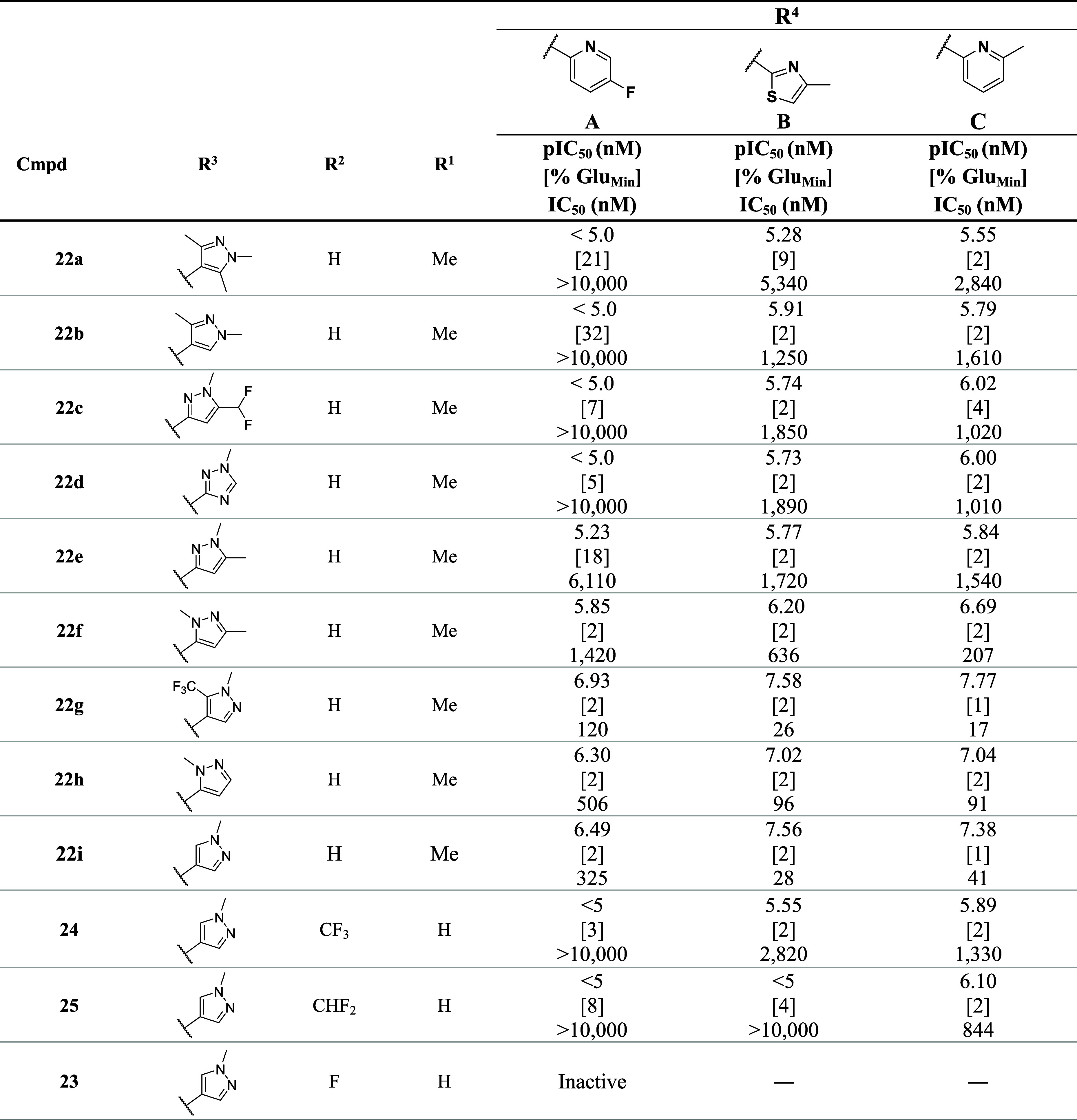
Structures and Activities for Analogs **22–25**^a^

a Calcium mobilization assays in human mGlu_5_-HEK293A cells were performed in the presence of an EC_80_ fixed concentration of glutamate, *n* = 2 independent experiments in triplicate. The % Glu_Min_ is the measure of efficacy of the NAM to reduce an EC_80_ response of glutamate.

Preparation of intermediate **16** began with commercially available iodide **29**, which underwent an Ullmann biaryl ether formation in the presence of alcohol **27** to afford ether **30** (Scheme 3). A subsequent palladium-catalyzed carbonylation provided ethyl ester **16**. Next, the synthesis of intermediate **17** began with a Wohl–Ziegler bromination of commercially available ester **31** to yield *gem*-dibromide **32** (Scheme 4). Geminal halide hydrolysis of intermediate **32** using AgNO_3_ as the oxidizing agent provided aldehyde **33**, which could undergo further transformation with diethylaminosulfur trifluoride (DAST) to give the difluoro intermediate **34**. Utilizing standard S_N_Ar conditions to react intermediate **34** with alcohol **27** afforded intermediate **17**. Saponification of esters **16** and **17** to carboxylic acids **20** and **21**, respectively, proceeded smoothly in near quantitative yields. Finally, conversion to the acid chloride and reaction with various heterocyclic amines *in situ* afforded analogs **24** and **25**.

**Scheme 3:**
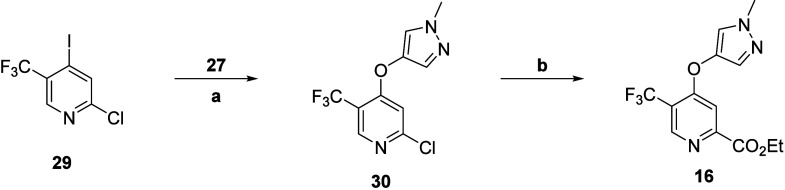
Synthesis of mGlu_5_ NAM Intermediate **16** Reagents and conditions: (a) CuI, Cs_2_CO_3_, DMF, μW 150 °C, 40%; (b) CO_(g)_, NaOAc, Pd(dppf)Cl_2_·CH_2_Cl_2_, EtOH/H_2_O (5:1), 70 °C, 99%.

**Scheme 4:**
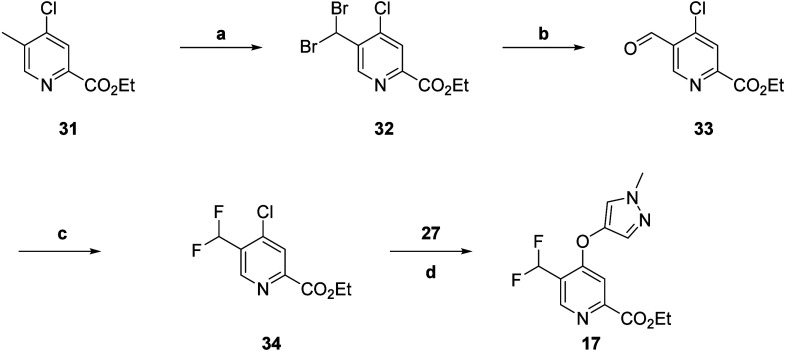
Synthesis of mGlu_5_ NAM Intermediate **17** Reagents and conditions: (a) NBS, AIBN, CCl_4_, 90 °C, 63%; (b) AgNO_3_, EtOH/H_2_O (10:1), 50 °C, 99%; (c) DAST, DCM, 53%; (d) Cs_2_CO_3_, DMF, μW 150 °C, 22%.

Select analogs **22**–**25** were screened against human mGlu_5_ (hmGlu_5_) to determine potency, with results highlighted in Table 1. These results emphasize the importance of the amide tail (**R^4^**). For instance, when the 5-fluoropyridine amide tail was installed (**22aA–22dA**), the hmGlu_5_ IC_50_’s were >10 μM; however, when the amide tail was exchanged for a 4-methylthiazole amide tail (**22aB–22dB**) or 6-methylpyridine (**22aC–22dC**), we observed hmGlu_5_ IC_50_’s = 1–5 μM. Moreover, it became evident with further SAR development that the combination of amide tail (**R**^**4**^) and 5-membered heteroaryl ether (**R**^**3**^) was crucial for activity. For example, while the 5-fluoropyridine amide tail provided several analogs with hmGlu_5_ IC_50_’s > 10 μM (**22a–dA**, **24**, and **25**), several analogs containing alternate heteroaryl ethers had IC_50_’s ≤ 500 nM (**22hA**, hmGlu_5_ IC_50_ = 506 nM; **22iA**, hmGlu_5_ IC_50_ = 325 nM; and **22gA**, hmGlu_5_ IC_50_ = 120 nM). This phenomenon was also observed in the 4-methylthaizole series (**22aB**, hmGlu_5_ IC_50_ = 5.3 μM vs **22gB**, hmGlu_5_ IC_50_ = 26 nM) as well as the 6-methylpyridine series (**22aC**, hmGlu_5_ IC_50_ = 2.8 μM vs **22hC**, hmGlu_5_ IC_50_ = 91 nM).

With the exceptions of **22f** and **22g**, di- or trisubstituted 5-membered heteroaryl analogs (**22a**–**e**) only afforded compounds with hmGlu_5_ IC_50_’s ≥ 1 μM. Interestingly, comparing **22bC** (hmGlu_5_ IC_50_ = 1.6 μM) with a constitutional isomer **22fC** (hmGlu_5_ IC_50_ = 207 nM) gave a 7.8-fold increase in potency. Introduction of a trifluoromethyl electron-withdrawing group to the 1-methyl-1*H*-pyrazole (**22gA**, hmGlu_5_ IC_50_ = 120 nM) resulted in a ∼3-fold increase in potency in the context of the 5-fluoropyridine amide tail when compared to **22iA** (hmGlu_5_ IC_50_ = 325 nM); however, this modification had no effect on potency when comparing analogs with the 4-methylthaizole amide tail (**22gB**, hmGlu_5_ IC_50_ = 26 nM vs **22iB**, hmGlu_5_ IC_50_ = 28 nM). It was also noted that analogs **22iA-C** were generally more potent than regioisomers **22hA-C**; however, the changes in potency varied with the amine tail (**22iA** vs **22hA**, 1.6-fold increase; **22iB** vs **22hB**, 3.4-fold increase).

Finally, we evaluated alternative picolinamide cores (**23**–**25**). Exchanging the 6-methylpicolinamide core (**22iA**; hmGlu_5_ IC_50_ = 325 nM) to a 5-fluoropicolinamide core (**23A**) resulted in a complete loss of activity. While the 5-(trifluoromethyl)picolinamide core was tolerated, only micromolar potencies could be achieved (**24B**, hmGlu_5_ IC_50_ = 2.8 μM and **24C**, hmGlu_5_ IC_50_ = 1.3 μM). Additionally, the 5-(difluoromethyl)picolinomide core was tolerated only with the 6-methylpyrdine tail (**25C**, hmGlu_5_ IC_50_ = 844 nM). These results highlight the significance of the 6-methylpicolinamide core.

Of these compounds, **22f-C**, **22gA-B**, **22hB-C**, and **22iA-C** were advanced into a battery of *in vitro* DMPK assays and our standard rat plasma:brain level (PBL) cassette paradigm (Table 2).^41,42^ Regarding physicochemical properties, these analogs all possessed molecular weights less than 450 Da, with **22gA**, **22gB**, **22hB**, and **22iB** having the most attractive CNS xLogP values (2.07–3.01). Analogs **22fC**, **22gB**, **22hC**, and **22iC** displayed high human and rat predicted hepatic clearance (CL_hep_) based on microsomal CL_int_ data (human CL_hep_ > 15 mL/min/kg; rat CL_hep_ > 46 mL/min/kg); however, analogs **22gA** and **22iB** were predicted to have moderate human and rat hepatic clearance (human CL_hep_ of 7 and 14 mL/min/kg, rat CL_hep_ of 38 and 27 mL/min/kg, respectively). Interestingly, **22hB** was predicted to have moderate rat hepatic clearance (CL_hep_ = 27 mL/min/kg) but high human hepatic clearance (CL_hep_ = 19 mL/min/kg). Analog **22iA** provided the best predicted hepatic clearance profile, with low human (CL_hep_ = 6 mL/min/kg) and moderate rat (CL_hep_ = 28 mL/min/kg) clearances.

**Table 2:** *In Vitro* DMPK and Rat PBL Data for Select Analogs **22fC**, **22gA–C**, **22hB–C**, and **22iA–C**

	**22fC**	**22gA**	**22gB**	**22gC**	**22hB**	**22hC**	**22iA**	**22iB**	**22iC**
Property	VU6043937	VU6044946	VU6045093	VU6073906	VU6043657	VU6043658	VU6043653	VU6043654	VU6043655
MW	337.38	395.31	397.37	391.35	329.38	323.35	327.31	329.38	323.35
xLogP^a^	1.86	2.07	3.01	2.17	2.5	1.66	1.16	2.1	1.26
TPSA^a^	81.9	81.9	81.9	81.9	81.9	81.9	81.9	81.9	81.9
hmGlu_5_ IC_50_ (nM)	207	120	26	17	96	91	325	28	41

***In Vitro* PK Parameters**^b^									
CL_int_ (mL/min/kg), rat	436	82	817	600	45	320	48	44	234
CL_hep_ (mL/min/kg), rat	60	38	65	63	27	57	28	27	54
CL_int_ (mL/min/kg), human	71	11	70	68	216	241	9	46	77
CL_hep_ (mL/min/kg), human	16	7	16	16	19	19	6	14	17
Rat *f*_*u*__,plasma_	ND^d^	ND^d^	ND^d^	ND^d^	0.219	ND^d^	0.059	0.089	0.059
Human *f*_*u*__,plasma_	0.037	0.012	0.004	0.011	0.062	0.034	0.059	0.063	0.041
Rat *f*_*u*__,brain_	0.008	0.002	0.003	0.005	0.029	0.021	0.012	0.014	0.013

**Brain Distribution (0.25 h) (SD Rat; 0.2 mg/kg IV)**^c^									
*K*_p, brain:plasma_	1.02	3.08	5.57	2.98	1.13	2.42	1.68	1.37	1.04
K_p,uu, brain:plasma_	ND^d^	ND^d^	ND^d^	ND^d^	0.15	ND^d^	0.34	0.22	0.23

a TPSA and xLogP were calculated using Dotmatics platform.

b *f*_*u*_ = fraction unbound; equilibrium dialysis assay; brain = rat brain homogenates;

c *K*_p_ = total brain-to-plasma partition ratio; *K*_p,uu_ = unbound brain-to-plasma partition ratio [(brain *f*_*u*_ × total brain)/(plasma *f*_*u*_ × total plasma)].

d ND = not determined; samples had low analyte peaks, possibly unstable in rat plasma.

Of the compounds tested, only **22gB** displayed high protein binding to human plasma with unbound fraction (*f*_u,plasma_) < 0.01. Conversely, the best human plasma binding profiles belonged to compounds **22hB** and **22iA-C** (*f*_u,plasma_ > 0.04). Analogs **22fC**, **22gA**, and **22gB** were highly bound to rat brain homogenates (*f*_u,brain_ < 0.01) and were determined to possibly be unstable in rat plasma. By contrast, compounds **22hB** (*f*_u,brain_ = 0.029), **22hC** (*f*_u,brain_ = 0.021), and **22iA-C** (*f*_u,brain_ = 0.012–0.014) were moderately bound to rat brain homogenates. Although **22hC** was determined to potentially be unstable in rat plasma, analogs **22hB** and **22iA-C** displayed a high free fraction in rat plasma (*f*_u,plasma_’s > 0.04). All analogs tested were determined to have excellent CNS penetration (rat brain:plasma *K*_p_ ≥ 1.0); however, compound **22iA** displayed the best CNS distribution of unbound drug (*K*_p,uu_ = 0.34). The moderate CNS distribution of unbound drug of **VU6043653** is likely due to moderate binding to brain homogenate (*f*_u,brain_ = 0.012). **VU6043653** (**22iA**) gave the best overall DMPK profile and was selected for further characterization.

When evaluated for a full mGlu selectivity profile in functional assays, **VU6043653** (**22iA**) displayed high subtype selectivity across the mGlu receptors (mGlu_1_, mGlu_2_, mGlu_4_, mGlu_7_, and Glu_8_ = inactive; mGlu_3_ > 10 μM) (Table 3). Additionally, **VU6043653** displayed an excellent cytochrome (CYP) P450 inhibition profile, with IC_50_’s ≥ 30 μM across all isoforms tested (1A2, 2D6, 2C9, and 3A4). Highlighted in Table 4 are the *in vivo* rat PK parameters. **VU6043653** displayed 40% oral bioavailability at a 10 mg/kg dose and moderate plasma clearance (41 mL/min/kg) in rats. The volume of distribution was moderate (2.0 L/kg), indicating minimal tissue binding, and elimination *t*_1/2_ was ∼45 min. With promising rat PK in hand, **VU6043653** was progressed into higher species *in vivo* PK studies (Table 4). **VU6043653** displayed moderate oral bioavailability (20% at a 3 mg/kg dose) in dogs; however, suprahepatic plasma clearance (38 mL/min/kg) halted further progress toward clinical candidate status.

**Table 3:** Further *In Vitro* Characterization of **VU6043653** (**21iA)**

**Metabotropic Glutamate Selectivity**		
	IC_50_ (nM)	[%Glu_Min_]
human mGlu_1_^a^	inactive	
human mGlu_2_^b^	inactive	
human mGlu_3_^b^	>10,000	[58]
human mGlu_4_^a^	inactive	
human mGlu_7_^a^	inactive	
human mGlu_8_^a^	inactive	

**P450 Inhibition IC_**50**_ (μM)**^c^			
1A2	2D6	2C9	3A4
>30	>30	>30	>30

a Calcium mobilization assay.

b G-protein-gated inwardly rectifying potassium channel (GIRK) assay.

c Assay performed in pooled human liver microsomes (HLM) in the presence of NADPH with CYP-specific probe substrates.

**Table 4:**
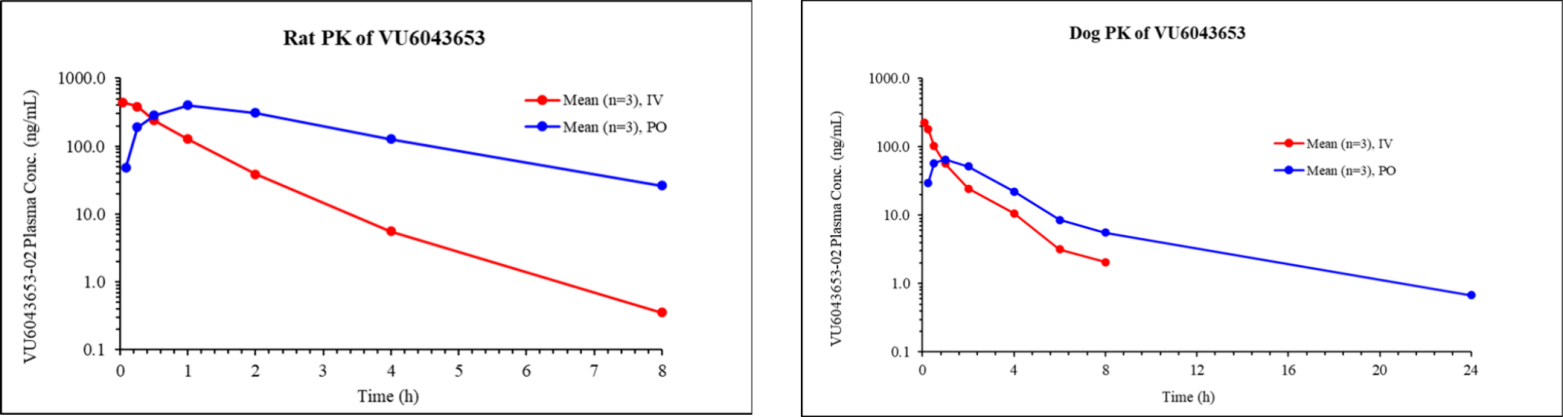
*In Vivo* Rat and Dog Pharmacokinetics of **VU6043653**

**IV PK**					
Species	Dose (mg/kg)	*t*_1/2_ (h)^b^	MRT (h)^b^	CL_p_ (mL/min/kg)^b^	*V*_ss_ (L/kg)^b^
Rat (SD)^a^	1.0	0.74	0.84	41	2.0
Dog (beagle)^c^	0.5	1.55	1.41	38	3.2

**PO PK**					
Species	Dose (mg/kg)	*T*_max_ (h)^e^	*C*_max_ (ng/mL)^e^	AUC_0-∞_ (h·ng/mL)^e^	%*F*^e^
Rat (SD)^d^	10.0	1.95	402	1320	43
Dog (beagle)^f^	3.0	0.67	70.3	264	20

a Male Sprague–Dawley rats (*n* = 3); vehicle = 10% ethanol, 70% PEG400, 20% saline.

b *t*_1/2_ = terminal phase plasma half-life; MRT = mean residence time; *V*_ss_ = volume of distribution at steady-state; CL_p_ = plasma clearance.

c Male beagle dogs (*n* = 3); vehicle = 10% ethanol, 70% PEG400, 20% saline.

d Male Sprague–Dawley rats (*n* = 3); vehicle = 0.5% aqueous methylcellulose with 0.1% Tween 80.

e *T*_max_ = time at which *C*_max_ occurs; *C*_max_ = maximum concentration; AUC = area under the curve; %*F* = oral bioavailability.

f Male beagle dogs (*n* = 3); 0.5% aqueous methylcellulose with 0.1% Tween 80 in saline.

Nonetheless, as a non-aryl/heterobiaryl acetylene mGlu_5_ NAM with an encouraging *in vivo* rodent PK profile, we wished to further assess **VU6043653** as a novel chemotype. Therefore, we compared metabolites in multiple species to better understand species differences in clearance and metabolism. These metabolism experiments, utilizing cryopreserved hepatocytes, identified amide hydrolysis as a major metabolite across all species tested (rats, dogs cynomolgus monkeys, and humans). Consistent with the high plasma clearance observed in dogs, high turnover was observed more so in dog hepatocytes than any other species tested (see the Supporting Information for additional details and results). To further evaluate our novel chemotype, the off-target and safety/toxicity profiles for this compound were further investigated. An ancillary pharmacology screen (Eurofins Panlabs)^38^ revealed both Adenosine A_3_ and Androgen receptors as potential off-target liabilities (≥70% inhibition at 10 μM) (see the Supporting Information for the full ancillary pharmacology profile).

In conclusion, we have established that 5-membered heterocycles are able to serve as competent isosteres for the metabolically labile pyrimidine of clinical candidate **VU0424238** (**10**) and predecessor compounds **11**. Of analogs assessed, **VU6043653** (**22iA**) displayed the best overall PK profile, with low human predicted hepatic clearance (CL_hep_ = 6 mL/min/kg), favorable rat and human plasma protein binding (*f*_u,plasma_ = 0.059), and high brain penetration (*K*_p_ = 1.68; *K*_p,uu_ = 0.34). **VU6043653** displayed high selectivity for mGlu_5_ over all other mGlu receptors evaluated (mGlu_1–4_ and mGlu_7–8_) and provided an improved CYP inhibition profile (CYP 2C9, 2D6, 3A4 IC_50_’s ≥ 30 μM) when compared to predecessor compounds **11**. In fact, **VU6043653** addressed many other challenges associated with compounds **11**, such as high predicted human CL_hep_, poor *f*_u_, and DAT inhibition. However, **VU6043653** did not progress forward due to its moderate potency in inhibiting human mGlu_5_ as well as poor higher species PK. Although this exercise did not provide mGlu_5_ NAMs with suitable DMPK profiles to warrant further advancement, it did highlight SAR insights for future scaffold designs. These refinements will be reported in due course.

## Supporting Information Available

General methods for the synthesis and characterization for key compounds and experimental details for calcium mobilization assays, *in vitro* and *in vivo* DMPK protocols, multispecies hepatocyte metabolism studies, and off-target assessment (PDF)

## Abbreviations

AO: Aldehyde oxidase
C_Lhep_: Hepatic clearance
CL_int_: Intrinsic clearance
CNS: Central nervous system
CYP: Cytochrome P450
DAT: Dopamine transporter
DMPK: Drug metabolism and pharmacokinetics
GIRK: G-protein-gated inwardly rectifying potassium channel
MAO-B: Monoamine oxidase-B
hmGlu_5_: Human metabotropic glutamate receptor subtype 5
mGluR: Metabotropic glutamate receptor
NAM: Negative allosteric modulator
SAR: Structure–activity relationship
