# Supplementary material for: Discovery of 4-(5-Membered)Heteroarylether-6-methylpicolinamide Negative Allosteric Modulators of Metabotropic Glutamate Receptor Subtype 5
Source: ACS Med Chem Lett. 2024 Nov 18;15(12):2210–9. doi: 10.1021/acsmedchemlett.4c00481 (PMC11647725; doi:10.1021/acsmedchemlett.4c00481)
Supplement: Supplementary file 1 — ml4c00481_si_001.pdf [file ml4c00481_si_001.pdf]

Supplemental Information for

Discovery of 4-(5-Membered)Heteroarylether-6-methylpicolinamide Negative Allosteric Modulators of Metabotropic Glutamate Receptor Subtype 5

Elizabeth S. Childress[a,b], Rory A. Capstick[a,b], , Katherine E. Crocker[a,b], Miranda L. Ledyard[a,b], Aaron M. Bender[a,b], Mallory A. Maurer[a,b], Natasha B. Billard[a,b], Hyekyung P. Cho[a,b], Alice L. Rodriguez[a,b], Colleen M. Niswender[a,b,d,e], Weimin Peng[b], Jerri M. Rook[b], Sichen Chang[a,b], Anna L. Blobaum[a,b], Olivier Boutaud[a,b], Analisa Thompson Gray[a,b], Carrie K. Jones[a,b], P. Jeffrey Conn[a,b,f], Andrew S. Felts[a,b] Craig W. Lindsley[a,b,c,d*], Kayla J. Temple[a,b*]

[a]Warren Center for Neuroscience Drug Discovery, Vanderbilt University, Nashville, TN 37232, USA
[b]Department of Pharmacology, Vanderbilt University School of Medicine, Nashville, TN 37232, USA
[c]Department of Chemistry, Vanderbilt University, Nashville, TN 37232, USA
[d]Department of Biochemistry, Vanderbilt University, Nashville, TN 37232, USA
[e]Vanderbilt Kennedy Center, Vanderbilt University School of Medicine, Nashville, TN 37232, USA
[f]Vanderbilt Brain Institute, Vanderbilt University School of Medicine, Nashville, TN 37232, USA
*Corresponding authors' email:
kayla.temple@vanderbilt.edu
craig.lindsley@vanderbilt.edu

Table of Contents

Experimental Synthetic Procedures and Spectroscopic Data S2
General Synthetic Methods. S2
General Instrumentation Methods. S2
General Procedure for the Preparation of Analogs 22iA-C. S3
General Procedure for the Preparation of Analog 22fC. S6
General Procedure for the Preparation of Analogs 22gA-C. S8
General Procedure for the Preparation of Analogs 22hB & 22hC. S11
Molecular Pharmacology Methods S14
Calcium Mobilization Assays S14
DMPK Methods S16
IV plasma-brain level determination (PBL). S16
Binding in plasma from rat and human. S17
Binding in brain homogenate from rat. S18
Intrinsic Clearance in Rat and Human Liver Microsomes S18
LC-MS/MS Analysis S19
Ancillary Pharmacology S21

Experimental Synthetic Procedures and Spectroscopic Data

General Synthetic Methods.

All reactions were carried out employing standard chemical techniques. Solvents used for extraction, washing, and chromatography were HPLC grade. All reagents were purchased from commercial sources and were used without further purification.

Automated flash column chromatography was performed on a Biotage Isolera 1 or a Teledyne ISCO CombiFlash system. RP-HPLC was performed on a Gilson preparative reversed-phase HPLC system comprised of a 333 aqueous pump with solvent-selection valve, 334 organic pump, GX-271 or GX-281 liquid hander, two column switching valves, and a 155 UV detector. Absorbance was typically monitored at 215 or 220 nm. Column: Phenomenex Axia-packed Gemini C18, 5 µm. Mobile phase: CH_3CN in H_2O (0.1% TFA) or CH_3CN in H_2O (0.05% *v/v* NH_4OH) under the specified gradient, then hold 95% CH_3CN in 5% aqueous phase, 50 mL/min, 23° C. All compounds were found to be >95% pure by LCMS analysis.

***Safety statement**: no unexpected or unusually high safety hazards were encountered.*

General Instrumentation Methods.

All NMR spectra were recorded on a 400 MHz AMX Bruker NMR spectrometer. ^1H and ^{13}C chemical shifts are reported in δ values in ppm downfield with the deuterated solvent as the internal standard. Data are reported as follows: chemical shift, multiplicity (s = singlet, d = doublet, t = triplet, q = quartet, b = broad, m = multiplet), integration, coupling constant (Hz).

Low resolution mass spectra (LRMS) were obtained on an Agilent 6120/6150 or Waters QDa (Performance) SQ MS with ESI source. *Method A (Agilent 6120/6150):* MS parameters were as follows: fragmentor: 70, capillary voltage: 3000 V, nebulizer pressure: 30 psig, drying gas flow: 13 L/min, drying gas temperature: 350 ºC. Samples were introduced via an Agilent 1290 UHPLC comprised of a G4220A binary pump, G4226A ALS, G1316C TCC, and G4212A DAD with ULD flow cell. UV absorption was generally observed at 215 nm and 254 nm with a 4 nm bandwidth. Column: Waters Acquity BEH C18, 1.0 x 50 mm, 1.7 um. Gradient conditions: 5% to 95% CH_3CN in H_2O (0.1% TFA) over 1.4 min, hold at 95% CH_3CN for 0.1 min, 0.5 mL/min, 55 ºC. *Method B (Agilent 6120/6150):* MS parameters were as follows:

fragmentor: 100, capillary voltage: 3000 V, nebulizer pressure: 40 psig, drying gas flow: 11 L/min, drying gas temperature: 350 ºC. Samples were introduced via an Agilent 1200 HPLC comprised of a degasser, G1312A binary pump, G1367B HP-ALS, G1316A TCC, G1315D DAD, and a Varian 380 ELSD (if applicable). UV absorption was generally observed at 215 nm and 254 nm with a 4 nm bandwidth. Column: Thermo Accucore C18, 2.1 x 30 mm, 2.6 um. Gradient conditions: 7% to 95% CH_3CN in H_2O (0.1% TFA) over 1.6 min, hold at 95% CH_3CN for 0.35 min, 1.5 mL/min, 45 ºC. *Method C (Waters QDa (Performance) SQ MS)*: MS parameters were as follows: cone voltage: 15 V, capillary voltage: 0.8 kV, probe temperature: 600° C. Samples were introduced via an Acquity I-Class PLUS UPLC comprised of a BSM, FL-SM, CH-A, and PDA. UV absorption was generally observed at 215 nm and 254 nm; 4 nm bandwidth. Column: Phenomenex EVO C18, 1.0 x 50 mm, 1.7 um. Column temperature: 55° C. Flow rate: 0.4 mL/min. Default gradient: 5% to 95% CH_3CN (0.05% TFA) in H_2O (0.05% TFA) over 1.4 min (curve 6), hold at 95% CH_3CN for 0.1 min. "Polar" (2% to 70% CH_3CN (0.05% TFA) in H_2O (0.05% TFA) over 0.8 min (curve 6), transition to 95% CH_3CN over 0.1 min (curve 6), hold at 95% CH_3CN for 0.6 min.) and "Non-Polar" (40% to 95% CH_3CN (0.05% TFA) in H_2O (0.05% TFA) over 1.4 min (curve 6), hold at 95% CH_3CN for 0.1 min.) gradients were also available. *Method D (Waters QDa (Performance) SQ MS):* MS parameters were as follows: cone voltage: 15 V, capillary voltage: 0.8 kV, probe temperature: 600° C. Samples were introduced via an Acquity I-Class PLUS UPLC comprised of a BSM, FL-SM, CH-A, and PDA. UV absorption was generally observed at 215 nm and 254 nm with a 4 nm bandwidth. Column: Phenomenex EVO C18, 1.0 x 50 mm, 1.7 um. Column temperature: 55° C. Flow rate: 0.4 mL/min. Default gradient: 5% to 95% CH_3CN in H_2O (5 mM NH_4HCO_3) over 1.4 min (curve 6), hold at 95% CH_3CN for 0.1 min. "Polar" (2% to 70% CH_3CN in H_2O (5 mM NH_4HCO_3) over 0.8 min (curve 6), transition to 95% CH_3CN over 0.1 min (curve 6), hold at 95% CH_3CN for 0.6 min.) and "Non-Polar" (40% to 95% CH_3CN in H_2O (5 mM NH_4HCO_3) over 1.4 min (curve 6), hold at 95% CH_3CN for 0.1 min.) gradients were also available.

High resolution mass spectra (HRMS) were obtained on an Agilent 6540 UHD Q-TOF with ESI source. MS parameters were as follows: fragmentor: 150, capillary voltage: 3500 V, nebulizer pressure: 60 psig, drying gas flow: 13 L/min, drying gas temperature: 275 ºC. Samples were introduced via an Agilent 1200 UHPLC comprised of a G4220A binary pump, G4226A 3 ALS, G1316C TCC, and G4212A DAD with ULD flow cell. UV absorption was observed at 215 nm and 254 nm with a 4 nm bandwidth. Column: Agilent Zorbax Extend C18, 1.8 µm, 2.1 x 50 mm. Gradient conditions: 5% to 95% CH_3CN in H_2O (0.1% formic acid) over 1 min, hold at 95% CH_3CN for 0.1 min, 0.5 mL/min, 40 ºC.

General Procedure for the Preparation of Analogs 22iA-C.

*Synthesis of Intermediates **14i** (Scheme 1):*

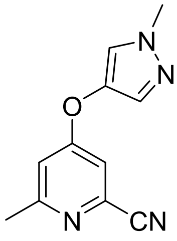

14i

4-Chloro-6-methylpicolinonitrile (100 mg, 0.66 mmol), 1-methyl-1H-pyrazol-4-ol (77 mg, 0.79 mmol), K_2CO_3 (184 mg, 1.3 mmol) and DMF (0.7 mL) were added to a microwave vial. The vial was sealed and the mixture was irradiated in a microwave at 150 °C for 15 minutes. The mixture was then left to stir overnight at 50 °C on benchtop. After cooling, the reaction mixture was diluted with water and extracted with DCM (3x). The combined organics were passed through a phase separator and concentrated. Purification via normal-phase flash chromatography on silica gel (0 – 60% EtOAc/hexanes) afforded 96 mg (68% yield) of the title compound. ^{1}H NMR (400 MHz, DMSO-d_6) δ 7.88 (s, 1H), 7.53 (d, J = 2.2 Hz, 1H), 7.47 (s, 1H), 7.16 (d, J = 2.2 Hz, 1H), 3.83 (s, 3H), 2.45 (s, 3H); LRMS: $C_{11}H_{10}N_4O$ $[M+H]^+$ calc. mass 215.1, found 215.4.

*Synthesis of Intermediate **18i** (Scheme 1):*

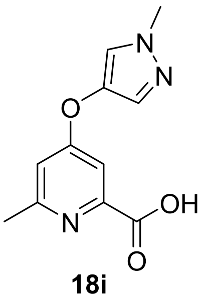

18i

To a solution of intermediate **14i** (96 mg, 0.45 mmol) in ethanol (0.9 mL) was added 2*N* NaOH (1.1 mL). The mixture was heated at 100 °C in a sealed vial for 1 hour. After cooling, the pH of the reaction was adjusted to pH 2-3 using 1*N* HCl and the mixture was concentrated *in vacuo*. The crude residue was dissolved in 10% MeOH/CH_2Cl_2, filtered to remove the insoluble salts, and the filtrate concentrated *in vacuo* to afford 104 mg (99% yield) of the title compound which was used without further purification. ^{1}H NMR (400 MHz, CD_3OD) δ 7.78 (s, 1H), 7.69 (d, J = 2.5 Hz, 1H), 7.50 (s, 1H), 7.34 (d, J = 2.6 Hz, 1H), 3.93 (s, 3H), 2.68 (s, 3H); LRMS: $C_{11}H_{11}N_3O_3$ $[M+H]^+$ calc. mass 234.1, found 234.2.

*Preparation of Compound **22iA (VU6043653)** (Scheme 1):*

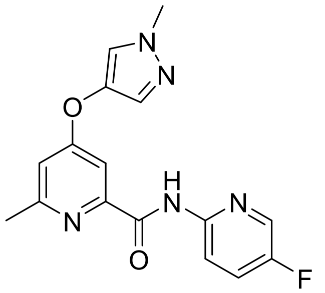

22iA

Intermediate **18i** (10 mg, 0.043 mmol) and 5-fluoro-2-aminopyridine (9.6 mg, 0.086 mmol) were dissolved in pyridine (1 mL) then cooled to 0 °C. Phosphorus(V) oxychloride (27 µL, 0.29 mmol) was added dropwise and the reaction was allowed to warm to room temperature over 30 minutes. The reaction was slowly added to a saturated aqueous $NaHCO_3$ solution at 0 °C. The mixture was extracted with chloroform:IPA (3:1) (3x) and the combined organic layers were dried ($MgSO_4$), filtered and concentrated *in vacuo*. Crude product was dissolved in DMSO (1 mL) and purified using RP-HPLC (25 - 85% ACN/0.1% aqueous TFA). Fractions containing product were basified with a saturated aqueous $NaHCO_3$ solution and extracted with EtOAc (3x). Solvents were concentrated to give 5.6 mg (40% yield) of the title compound. ^{1}H NMR (400 MHz, $CDCl_3$) δ 10.59 (s, 1H), 8.42 (dd, J = 9.1 Hz, J_{HF} = 4.1 Hz, 1H), 8.22 (d, J = 3.0 Hz, 1H), 7.68 (d, J = 2.3 Hz, 1H), 7.49 (ddd, J = 9.1, 3.0 Hz, J_{HF} = 7.6 Hz, 1H), 7.38 (d, J = 0.8 Hz, 1H), 7.34 (d, J = 0.8 Hz, 1H), 6.93 (d, J = 2.3 Hz, 1H), 3.93 (s, 3H), 2.56 (s, 3H); ^{13}C NMR (101 MHz, $CDCl_3$) δ 167.50, 162.26, 159.74, 156.58 (d, J_{CF} = 251.2 Hz), 150.75, 147.56 (d, J_{CF} = 2.5 Hz), 137.87, 135.81 (d, J_{CF} = 25.4 Hz), 131.52, 125.37 (d, J_{CF} = 19.4 Hz), 121.66, 114.78 (d, J_{CF} = 4.3 Hz), 113.29, 107.23, 40.06, 24.40; HRMS: $C_{16}H_{14}FN_5O_2$ $[M+H]^+$ calc. mass 328.1204, found 328.1204.

*Preparation of Compound **22iB (VU6043654)** (Scheme 1):*

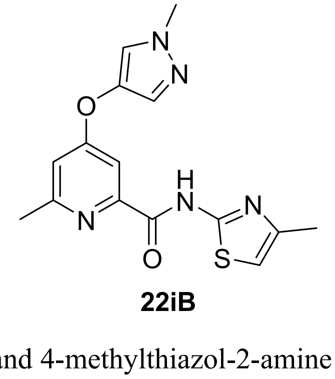

22iB

Intermediate **18i** (35 mg, 0.15 mmol) and 4-methylthiazol-2-amine (34 mg, 0.30 mmol) were dissolved in pyridine (0.5 mL) then cooled to 0 °C. Phosphorus(V) oxychloride (34 µL, 0.36 mmol) was added dropwise and the reaction was allowed to warm to room temperature and stirred for 17 hours. The reaction was slowly

added to a saturated aqueous $NaHCO_3$ solution at 0 °C. The mixture was extracted with chloroform:IPA (3:1) (3x) and the combined organic layers were passed through a phase separator and concentrated. Crude product was dissolved in DMSO (1 mL) and purified using RP-HPLC (25-55% ACN/0.5% aqueous NH_4OH). Fractions were concentrated to give 4.8 mg (10% yield) of the title compound. ^{1}H NMR (400 MHz, Acetone-d_6) δ 11.15 (s, 1H), 7.76 (s, 1H), 7.58 (d, J = 2.4 Hz, 1H), 7.42 (d, J = 0.9 Hz, 1H), 7.16 (d, J = 2.4 Hz, 1H), 6.78 (q, J = 1.0 Hz, 1H), 3.93 (s, 3H), 2.59 (s, 3H), 2.32 (d, J = 1.1 Hz, 3H); ^{13}C NMR (101 MHz, Acetone-d_6) δ 168.7, 162.3, 161.4, 157.4, 150.4, 148.9, 138.5, 131.6, 123.0, 114.5, 109.4, 107.8, 40.1, 24.3, 17.3; HRMS: $C_{15}H_{15}N_5O_2S$ $[M+H]^+$ 330.1019, found 330.1020.

*Preparation of Compound **22iC (VU6043655)** (Scheme 1):*

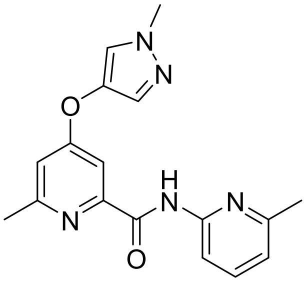

22iC

Intermediate **18i** (30 mg, 0.13 mmol) and 6-methylpyridin-2-amine (28 mg, 0.26 mmol) were dissolved in pyridine (1 mL) then cooled to 0 °C. Phosphorus(V) oxychloride (29 µL, 0.31 mmol) was added dropwise and the reaction was allowed to warm to room temperature over 2 hours. The reaction was slowly added to a saturated aqueous $NaHCO_3$ solution at 0 °C. The mixture was extracted with chloroform:IPA (3:1) (3x) and the combined organic layers were passed through a phase separator and concentrated. Purification via normal-phase column chromatography on silica gel (0 – 40% EtOAc/DCM) afforded 19 mg (45% yield) of title compound. ^{1}H NMR (400 MHz, $CDCl_3$) δ 10.44 (s, 1H), 8.19 (d, J = 8.2 Hz, 1H), 7.69 (d, J = 2.3 Hz, 1H), 7.63 (t, J = 7.9 Hz, 1H), 7.37 (s, 1H), 7.33 (s, 1H), 6.96 – 6.85 (m, 2H), 3.92 (s, 3H), 2.56 (s, 3H), 2.51 (s, 3H); ^{13}C NMR (101 MHz, $CDCl_3$) δ 167.41, 162.51, 159.66, 1z57.26, 151.10, 150.63, 138.71, 137.91, 131.54, 121.66, 119.52, 113.18, 110.98, 107.15, 40.04, 24.40, 24.32; HRMS: $C_{17}H_{17}N_5O_2$ $[M+H]^+$ 324.1455, found 324.1458.

General Procedure for the Preparation of Analog 22fC.

*Synthesis of Intermediate **14f** (Scheme 1):*

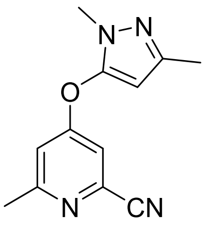

14f

A solution of 4-chloro-6-methylpicolinonitrile (100 mg, 0.66 mmol), 1,3-dimethyl-1*H*-pyrazol-5-ol (88 mg, 0.79 mmol) and potassium carbonate (184 mg, 1.3 mmol) in DMF (0.73 mL) was microwave irradiated to 150 °C for 15 minutes. The mixture was then left to stir overnight at 50 °C on benchtop. After cooling to ambient temperature, the mixture was diluted with water and extracted with DCM. The combined organics were passed through a phase separator and concentrated. Purification via normal-phase column chromatography on silica gel (0-70% EtOAc/hexanes) gave 67 mg (45% yield) of title compound. ^{1}H NMR (400 MHz, DMSO-d_6) δ 7.67 (d, J = 2.3 Hz, 1H), 7.27 (d, J = 2.3 Hz, 1H), 5.90 (s, 1H), 3.54 (s, 3H), 2.50 (s, 3H), 2.14 (s, 3H); LRMS: $C_{12}H_{12}N_4O$ $[M+H]^+$ calc. mass 229.1, found 229.2.

*Synthesis of Intermediate **18f** (Scheme 1):*

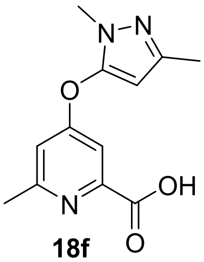

18f

To a solution of intermediate **14f** (67 mg, 0.29 mmol) in ethanol (0.6 mL) was added 2*N* NaOH (730 µL, 1.5 mmol). The mixture was heated at 100 °C in a sealed vial for 1 hour. After cooling, the pH of the reaction was adjusted to pH 2-3 using 1*N* HCl and the mixture was concentrated *in vacuo*. The crude residue was dissolved in 10% MeOH/CH_2Cl_2, filtered to remove the insoluble salts, and the filtrate concentrated *in vacuo* to afford 46 mg (64% yield) of the title compound which was used without further purification. ^{1}H NMR (400 MHz, CD_3OD) δ 7.62 (d, J = 2.4 Hz, 1H), 7.22 (d, J = 2.4 Hz, 1H), 5.87 (s, 1H), 3.62 (s, 3H), 2.61 (s, 3H), 2.24 (s, 3H); LRMS: $C_{12}H_{13}N_3O_3$ $[M+H]^+$ calc. mass 248.1, found 248.2.

*Preparation of Compound **22fC (VU6043937)** (Scheme 1):*

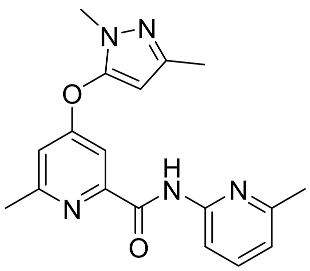

22fC

Intermediate **18f** (35 mg, 0.14 mmol) and 6-methylpyridin-2-amine (31 mg, 0.28 mmol) were dissolved in pyridine (0.5 mL) then cooled to 0 °C. Phosphorus(V) oxychloride (32 µL, 0.34 mmol) was added dropwise and the reaction was allowed to warm to room temperature and stirred for 17 hours. The reaction was slowly added to a saturated aqueous $NaHCO_3$ solution at 0 °C. The mixture was extracted with chloroform:IPA (3:1) (3x) and the combined organic layers were passed through a phase separator and concentrated. Crude product was dissolved in DMSO (1 mL) and purified using RP-HPLC (35-65% ACN/0.5% aqueous NH_4OH). Fractions were concentrated to give 7.2 mg (15% yield) of title compound. ^{1}H NMR (400 MHz, DMSO-d_6) δ 10.30 (s, 1H), 8.05 (d, J = 8.2 Hz, 1H), 7.78 (dd, J = 7.9, 7.9 Hz, 1H), 7.55 (d, J = 2.5 Hz, 1H), 7.31 (d, J = 2.3 Hz, 1H), 7.08 (d, J = 7.5 Hz, 1H), 5.92 (s, 1H), 3.56 (s, 3H), 2.62 (s, 3H), 2.45 (s, 3H), 2.17 (s, 3H); ^{13}C NMR (101 MHz, DMSO-d_6) δ 164.6, 161.1, 160.6, 157.2, 150.5, 149.6, 146.8, 146.3, 139.0, 119.7, 113.9, 110.0, 106.8, 92.6, 33.9, 23.9, 23.6, 14.2; HRMS: $C_{18}H_{19}N_5O_2$ $[M+H]^+$ calc. mass 338.1612, found 338.1613.

General Procedure for the Preparation of Analogs 22gA-C.

*Synthesis of Intermediate **14g** (Scheme 1):*

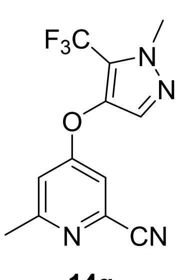

14g

To a vial was added 4-chloro-6-methylpicolinonitrile (100 mg, 0.66 mmol), 1-methyl-5-(trifluoromethyl)-1*H*-pyrazol-4-ol (65 mg, 0.39 mmol) and potassium carbonate (184 mg, 1.3 mmol) in DMF (0.7 mL). The

vial was sealed and heated on benchtop at 50 °C for 4 hours. The reaction mixture was cooled to room temperature and left to stir overnight. The mixture was the heated to 50 °C for an additional 2.5 hours. The reaction was cooled to ambient temperature, diluted with water, and extracted with DCM (3x). The combined organics were passed through a phase separator, concentrated, and via normal-phase column chromatography on silica gel purified (0 – 60% EtOAc/hexanes) to give 76 mg (69% yield) of title compound. ^{1}H NMR (400 MHz, DMSO-d_6) δ 7.78 (s, 1H), 7.68 (d, J = 2.4 Hz, 1H), 7.28 (d, J = 2.4 Hz, 1H), 4.00 (q, J_{HF} = 1.0 Hz, 3H), 2.48 (s, 3H); LRMS: $C_{12}H_9F_3N_4O$ $[M+H]^+$ calc. mass 283.1, found 283.0.

*Synthesis of Intermediate **18g** (Scheme 1):*

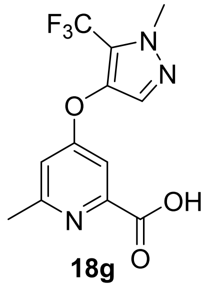

18g

To a solution of intermediate **14g** (76 mg, 0.27 mmol) in ethanol (0.5 mL) was added 2*N* NaOH (670 µL, 1.3). The mixture was heated at 100 °C in a sealed vial for 1 hour. After cooling, the pH of the reaction was adjusted to pH 2-3 using 1*N* HCl and the mixture was concentrated *in vacuo*. The crude residue was dissolved in 10% MeOH/CH_2Cl_2, filtered to remove the insoluble salts, and the filtrate concentrated *in vacuo* to afford 75 mg (92% yield) of the title compound which was used without further purification. ^{1}H NMR (400 MHz, CD_3OD) δ 7.54 (s, 1H), 7.39 (m, 1H), 6.91 – 6.88 (m, 1H), 4.06 – 3.99 (m, 3H), 2.52 (s, 3H); LRMS: $C_{12}H_{10}F_3N_3O_3$ $[M+H]^+$ calc. mass 302.1, found 302.1.

*Preparation of Compound **22gA (VU6044946)** (Scheme 1):*

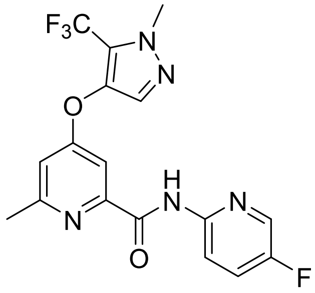

22gA

Intermediate **18g** (35 mg, 0.12 mmol) and 5-fluoro-2-aminopyridine (26 mg, 0.23 mmol) were dissolved in pyridine (0.5 mL) then cooled to 0 °C. Phosphorus(V) oxychloride (26 µL, 0.28 mmol) was added dropwise and the reaction was allowed to warm to room temperature and stirred for 17 hours. The reaction was slowly added to a saturated aqueous $NaHCO_3$ solution at 0 °C. The mixture was extracted with chloroform:IPA (3:1) (3x) and the combined organic layers were passed through a phase separator and concentrated. Crude product was dissolved in DMSO (1 mL) and purified using RP-HPLC (40-70% ACN/0.5% aqueous NH_4OH). Fractions were concentrated to give 7.9 mg (17% yield) of the title compound. ^{1}H NMR (400 MHz, Acetone-d_6) δ 10.48 (s, 1H), 8.41 (dd, J = 9.1 Hz, J_{HF} = 4.1 Hz, 1H), 8.30 (d, J_{HF} = 3.0 Hz, 1H), 7.77 – 7.70 (m, 1H), 7.68 (s, 1H), 7.60 (d, J = 2.4 Hz, 1H), 7.21 (d, J = 2.4 Hz, 1H), 4.09 (q, J_{HF} = 0.9 Hz, 3H), 2.62 (s, 3H); ^{13}C NMR (101 MHz, Acetone-d_6) δ 167.7, 162.2, 161.5, 157.6 (d, J_{CF} = 249.2 Hz), 151.9, 148.6 (d, J_{CF} = 2.0 Hz), 138.3 (q, J_{CF} = 1.7 Hz), 136.7 (d, J_{CF} = 25.6 Hz), 132.3, 126.2 (d, J_{CF} = 19.8 Hz), 122.6 (q, J_{CF} = 39.4 Hz), 121.7 (q, J_{CF} = 269.7 Hz), 115.1 (d, J_{CF} = 4.5 Hz), 114.2, 107.5, 40.1 (q, J_{CF} = 1.9 Hz), 24.3.; HRMS $C_{17}H_{13}F_4N_5O_2$ $[M+H]^+$ calc. mass 396.1078, found 396.1080.

*Preparation of Compound **22gB (VU6045093)** (Scheme 1):*

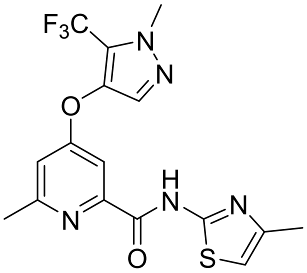

22gB

Intermediate **18g** (35 mg, 0.12 mmol) and 4-methylthiazol-2-amine (27 mg, 0.23 mmol) were dissolved in pyridine (0.5 mL) then cooled to 0 °C. Phosphorus(V) oxychloride (26 µL, 0.28 mmol) was added dropwise and the reaction was allowed to warm to room temperature and stirred for 17 hours. The reaction was slowly added to a saturated aqueous $NaHCO_3$ solution at 0 °C. The mixture was extracted with chloroform:IPA (3:1) (3x) and the combined organic layers were passed through a phase separator and concentrated. Crude product was dissolved in DMSO (1 mL) and purified using RP-HPLC (35-70% ACN/0.5% aqueous NH_4OH). Fractions were concentrated to give 17 mg (36% yield) of the title compound. ^{1}H NMR (400 MHz, Acetone-d_6) δ 11.18 (s, 1H), 7.68 (s, 1H), 7.56 (d, J = 2.4 Hz, 1H), 7.22 (d, J = 2.4 Hz, 1H), 6.78 (q, J = 1.0 Hz, 1H), 4.09 (q, J_{HF} = 0.9 Hz, 3H), 2.61 (s, 3H), 2.31 (d, J = 1.1 Hz, 3H); ^{13}C NMR (101 MHz, Acetone-d_6) δ 167.6, 162.2, 161.8, 157.4, 150.7, 148.9, 138.3 (q, J_{CF} = 1.7 Hz), 132.3, 122.6 (q, J_{CF} = 39.4

Hz), 120.9 (q, J_{CF} = 269.7 Hz), 114.5, 109.4, 107.8, 40.1 (q, J_{CF} = 2.0 Hz), 24.3, 17.3. HRMS: $C_{16}H_{14}F_3N_5O_2S$ $[M+H]^+$ calc. mass 398.0893, found 398.0890.

Preparation of Compound ***22gC (VU6073906)*** *(Scheme 1):*

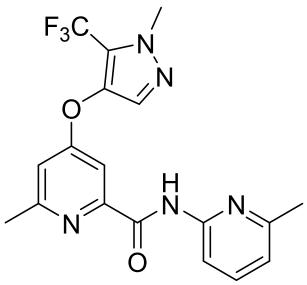

22gC

Intermediate **18g** (31 mg, 0.10 mmol) and 6-methylpyridin-2-amine (23 mg, 0.21 mmol) were dissolved in pyridine (0.7 mL) then cooled to 0 °C. Phosphorus(V) oxychloride (23 µL, 0.25 mmol) was added dropwise and the reaction was allowed to warm to room temperature over 2 hours. The reaction was slowly added to a saturated aqueous $NaHCO_3$ solution at 0 °C. The mixture was extracted with chloroform:IPA (3:1) (3x) and the combined organic layers were passed through a phase separator and concentrated. Purification via normal-phase column chromatography on silica gel (0 – 60% EtOAc/hexanes) afforded 20 mg (50% yield) of title compound. ^{1}H NMR (400 MHz, $CDCl_3$) δ 10.43 (s, 1H), 8.19 (d, J = 8.2 Hz, 1H), 7.74 – 7.57 (m, 2H), 7.41 (d, J = 0.8 Hz, 1H), 6.93 (d, J = 7.5 Hz, 1H), 6.91 (d, J = 2.4 Hz, 1H), 4.04 (q, J_{HF} = 0.8 Hz, 3H), 2.59 (s, 3H), 2.51 (s, 3H); ^{13}C NMR (101 MHz, $CDCl_3$) δ 166.54, 162.25, 159.97, 157.29, 151.36, 150.57, 138.72, 137.39 (q, J_{CF} = 1.5 Hz), 131.48, 122.58 (q, J_{CF} = 39.2 Hz), 119.65 (q, J_{CF} = 269.3 Hz), 119.59, 113.25, 111.00, 106.85, 39.63 (q, J_{CF} = 1.9 Hz), 24.41, 24.32; HRMS $C_{18}H_{16}F_3N_5O_2$ $[M+H]^+$ calc. mass 392.1329, found 392.1329.

<u>General Procedure for the Preparation of Analogs 22hB & 22hC.</u>

Synthesis of Intermediate ***14h*** *(Scheme 1):*

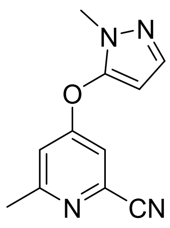

14h

To a microwave vial was added 4-chloro-6-methylpicolinonitrile (100 mg, 0.66 mmol), 1-methyl-1*H*-pyrazol-5-ol (88 mg, 0.79 mmol) and potassium carbonate (180 mg, 1.3 mmol) in DMF (0.7 mL). The vial was sealed and the mixture was irradiated in a microwave at 150 °C for 15 minutes. The mixture was then left to stir overnight at 50 °C on benchtop. After cooling, the reaction mixture was diluted with water and extracted with DCM (3x). The combined organics were passed through a phase separator and concentrated. Purification via normal-phase flash chromatography on silica gel (0 – 60% EtOAc/hexanes) afforded 73 mg (52% yield) of the title compound. ^{1}H NMR (400 MHz, DMSO-d_6) δ 7.68 (d, J = 2.3 Hz, 1H), 7.49 (d, J = 2.1 Hz, 1H), 7.27 (d, J = 2.3 Hz, 1H), 6.11 (d, J = 2.0 Hz, 1H), 3.63 (s, 3H), 2.50 (s, 3H); LRMS: $C_{11}H_{10}N_4O$ $[M+H]^+$ calc. mass 215.1, found 215.2.

Synthesis of Intermediate ***18h*** *(Scheme 1):*

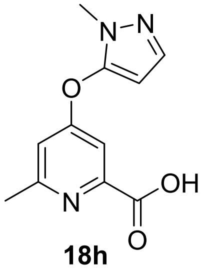

18h

To a solution of intermediate **14h** (67 mg, 0.29 mmol) in ethanol (0.6 mL) was added 2*N* NaOH (730 µL, 1.5 mmol). The mixture was heated at 100 °C in a sealed vial for 1 hour. After cooling, the pH of the reaction was adjusted to pH 2-3 using 1*N* HCl and the mixture was concentrated *in vacuo*. The crude residue was dissolved in 10% MeOH/CH_2Cl_2, filtered to remove the insoluble salts, and the filtrate concentrated *in vacuo* to afford 71 mg (90% yield) of the title compound which was used without further purification. ^{1}H NMR (400 MHz, CD_3OD) δ 7.48 (br d, J = 2.2 Hz, 2H), 7.01 (d, J = 2.4 Hz, 1H), 5.99 (d, J = 2.1 Hz, 1H), 3.69 (s, 3H), 2.55 (s, 3H); LRMS: $C_{11}H_{11}N_3O_3$ $[M+H]^+$ calc. mass 234.1, found 234.2.

Preparation of Compound ***22hB (VU6043657)*** *(Scheme 1):*

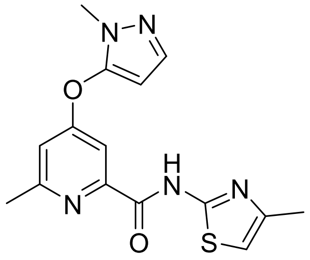

22hB

Intermediate **18h** (35 mg, 0.15 mmol) and 4-methylthiazol-2-amine (34 mg, 0.30 mmol) were dissolved in pyridine (0.5 mL) then cooled to 0 °C. Phosphorus(V) oxychloride (34 µL, 0.36 mmol) was added dropwise and the reaction was allowed to warm to room temperature and stirred for 17 hours. The reaction was slowly added to a saturated aqueous $NaHCO_3$ solution at 0 °C. The mixture was extracted with chloroform:IPA (3:1) (3x) and the combined organic layers were passed through a phase separator and concentrated. Crude product was dissolved in DMSO (1 mL) and purified using RP-HPLC (25-55% ACN/0.5% aqueous NH_4OH). Fractions were concentrated to give 5.6 mg (11% yield) of the title compound. ^1H NMR (400 MHz, Acetone-d_6) δ 11.18 (s, 1H), 7.64 (d, J = 2.4 Hz, 1H), 7.46 (d, J = 2.0 Hz, 1H), 7.26 (d, J = 2.4 Hz, 1H), 6.80 (q, J = 1.1 Hz, 1H), 6.05 (d, J = 2.0 Hz, 1H), 3.70 (s, 3H), 2.64 (s, 3H), 2.32 (d, J = 1.1 Hz, 3H); ^{13}C NMR (101 MHz, Acetone-d_6) δ 166.2, 162.1 (2C), 157.4, 150.8, 149.0, 148.2, 139.0, 114.9, 109.5, 108.4, 94.0, 34.9, 24.3, 17.3; HRMS: $C_{15}H_{15}N_5O_2S$ $[M+H]^+$ calc. mass 330.1019, found 330.1022.

*Preparation of Compound **22hC (VU6043658)** (Scheme 1):*

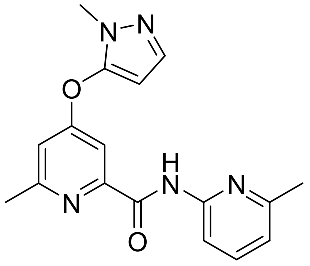

22hC

Intermediate **18h** (20 mg, 0.086 mmol) and 6-methylpyridin-2-amine (19 mg, 0.17 mmol) were dissolved in pyridine (0.5 mL) then cooled to 0 °C. Phosphorus(V) oxychloride (19 µL, 0.21 mmol) was added dropwise and the reaction was allowed to warm to room temperature and stirred for 17 hours. The reaction was slowly added to a saturated aqueous $NaHCO_3$ solution at 0 °C. The mixture was extracted with chloroform:IPA (3:1) (3x) and the combined organic layers were passed through a phase separator and concentrated. Crude product was dissolved in DMSO (1 mL) and purified using RP-HPLC (30-60% ACN/0.5% aqueous NH_4OH). Fractions were concentrated to give 10 mg (21% yield) of title compound. ^1H NMR (400 MHz, DMSO-d_6) δ 10.29 (s, 1H), 8.04 (d, J = 8.2 Hz, 1H), 7.78 (dd, J = 7.8, 7.8 Hz, 1H),

7.55 (d, J = 2.4 Hz, 1H), 7.52 (d, J = 2.0 Hz, 1H), 7.31 (d, J = 2.4 Hz, 1H), 7.07 (d, J = 7.7 Hz, 1H), 6.13 (d, J = 2.0 Hz, 1H), 3.65 (s, 3H), 2.62 (s, 3H), 2.44 (s, 3H); ^{13}C NMR (101 MHz, DMSO-d_6) δ 164.6, 161.1, 160.6, 157.2, 150.6, 149.6, 146.9, 139.0, 138.2, 119.7, 113.9, 110.0, 106.8, 93.4, 34.3, 23.9, 23.7; HRMS $C_{17}H_{17}N_5O_2$ $[M+H]^+$ calc. mass 324.1455, found 324.1459.

Molecular Pharmacology Methods

Calcium Mobilization Assays.

To measure the functional activity of negative allosteric modulator (NAM) compounds in a cellular assay, human metabotropic glutamate receptor subtype 5 ($mGlu_5$) was stably expressed in Human Embryonic Kidney (HEK293A) cells to evoke a decrease in intracellular calcium to an EC_{80} concentration of glutamate (Glu) agonist. Stably expressing $mGlu_5$-HEK293A cells were cultured in DMEM medium containing 10% fetal bovine serum, 20 mM HEPES, 2 mM glutamine, 1 mM sodium pyruvate, non-essential amino acid mixture, 0.5 mg/ml G418, and antibiotics/antimycotic. All reagents used were from Life Technologies (Carlsbad, CA) unless otherwise noted.

Briefly, the day before the assay, HEK293A cells stably expressing $mGlu_5$ (20,000 cells/20 µL/well) were plated in in black-walled, clear-bottomed, amine-treated 384 well plates (Corning) in the assay medium (DMEM containing 10% dialyzed fetal bovine serum, 20 mM HEPES, 1 mM sodium pyruvate, and antibiotics/antimycotic). Cells were incubated overnight at 37 °C in the presence of 5% CO_2. The next day, calcium assay buffer (Hank's balanced salt solution (HBSS), 20 mM HEPES, 2.5 mM Probenecid, 4.16 mM sodium bicarbonate (Sigma-Aldrich, St. Louis, MO)) was prepared to dilute compounds, agonists, and Fluo-4-acetomethoxyester (Fluo-4-AM, Ion Biosciences), fluorescent calcium indicator dye. Compounds were serially diluted 1:3 into 10-point concentration response curves in DMSO using a Bravo Liquid Handler (Agilent, Santa Clara, CA), transferred to a 384 well daughter plates using an Echo acoustic liquid handler (Beckman Coulter, Indianapolis, Indiana), and diluted in assay Buffer to a 2X final concentration. The agonist plates were prepared using glutamate (Tocris) concentrations for the EC_{20}, EC_{80} and EC_{MAX} responses by diluting in assay buffer to a 5X final concentration. A 2X dye solution (2.3 µM) was prepared by mixing a 2.3 mM Fluo-4-AM stock in DMSO with 10% (w/v) pluronic acid F-127 in a 1:1 ratio in assay buffer. Using a microplate washer (BioTek, Winooski, VT), cells were washed with assay buffer 3 times to remove media. After the final wash, 20 µL of assay buffer remained in the cell plates. 20 µL of the 2X dye solution (final 1.15 µM) was added to each well of the cell plate using a Multidrop Combi dispenser (Thermo Fisher, Waltham, MA). After cells were incubated with the dye solutions for 45 min at 37 °C in

the presence of 5% CO_2, the dye solutions were removed and replaced with assay buffer using a microplate washer, leaving 20 µL of assay buffer in the cell plate. The compound, agonist, and cell plates were placed inside the Functional Drug Screening System (FDSS 7000 or uCell, Hamamatsu, Japan) to measure the calcium flux. Assays were run at 37 °C. A triple add protocol was used to measure Ca kinetics. Briefly, after establishment of a fluorescence baseline for 2 seconds (excitation, 480 nm; emission, 530 nm), 20 µL of test compound was added to the cells and the response was measured for 140 seconds. This was followed by the addition of 10 µL (5X) of an EC_{20} concentration of Glu agonist,, and the response of the cells was measured for 125 seconds. A third addition occurred by adding 12 uL (5X) of an EC_{80} concentration of Glu agonist and the response of the cells was measured for 90 seconds. Vehicle (0.6 % DMSO) in assay buffer was added to the control wells at the 1st add to ensure vehicle matching for the glutamate EC_{20}, EC_{80}, and EC_{Max} additions. Calcium fluorescence was recorded as fold over basal fluorescence and raw data were normalized to the maximal response to Glu agonist (EC_{Max}). Compound-evoked decreases in calcium response in the presence of Glu EC_{80} agonist were determined as inhibition activity, and potency (IC_{50}) and maximum inhibition responses (% Glu_{Min}) of compounds were determined using a four-parameter logistical equation using GraphPad Prism (La Jolla, CA) or the Dotmatics software platform (Woburn, MA) :

$$y = bottom + \frac{top - bottom}{1 + 10^{(LogEC50 - A)Hillslope}}$$

where *A* is the molar concentration of the compound; *bottom* and *top* denote the lower and upper plateaus of the concentration-response curve; HillSlope is the Hill coefficient that describes the steepness of the curve; and IC_{50} is the molar concentration of compound required to generate a response halfway between the *top* and *bottom*.

mGlu Selectivity Assays:

The mGlu subtype selectivity of compounds was determined using calcium mobilization or thallium flux GIRK (G protein-regulated inwardly rectifying K channel) assays using the stable cell lines expressing the respective mGlu receptors. Calcium mobilization assay was performed for mGlu subtypes 1, 4, 7, and 8. Tetracycline-inducible human $mGlu_1$ in TREx-293 cells were used as previously described.[1] Human $mGlu_4$ stable cells co-expressing chimeric G_{qi5} proteins in Chinese Hamster Ovary (CHO) cells were used as previously described.[2] HEK293 cells stably expressing human $mGlu_7$ or $mGlu_8$ and the promiscuous G protein $G_{\alpha 15}$ (mouse) were maintained in growth medium containing 90% Dulbecco's Modified Eagle Media (DMEM), 10% fetal bovine serum (FBS), 100 units/mL penicillin/streptomycin, 20 mM HEPES, 1 mM sodium pyruvate, 2 mM L-glutamine, 0.1 mM non-essential amino acids, 700 µg/mL G418 sulfate, and 0.6 µg/mL puromycin. $mGlu_{7/8}$ cells were used as described above for $hmGlu_5$ with the following modifications. $mGlu_7$ cells were incubated with dye solution for 50 min at RT and FDSS assays were run

at RT using DL-AP4 for the agonist. $mGlu_8$ cells were incubated with dye solution for 50 min at 37 °C in the presence of 0.5% CO_2 and FDSS assays were run at RT. To measure activity at $mGlu_2$ and $mGlu_3$, HEK293-GIRK cells stably expressing human $mGlu_2$ and $mGlu_3$ were assessed using thallium flux assay through GIRK channels as previously described in detail.[3]

Human $mGlu_7/G_{a15}$/HEK cells (15,000 cells/20 μL/well) and human $mGlu_8/G_{a15}$/HEK cells (15,000 cells/20 μL/well) were plated in black-walled, clear-bottomed, TC-treated, 384 well plates (Greiner Bio-One, Monroe, NC) in DMEM containing 10% dialyzed FBS, 20 mM HEPES, 100 units/mL penicillin/streptomycin, and 1 mM sodium pyruvate (Plating Medium) as described in Jalan-Sakrikar et al., 2014. The cells were grown overnight at 37 °C in the presence of 5% CO_2.

DMPK Methods

IV plasma-brain level determination (PBL).

In-life phase

Compounds were formulated as a solution in ethanol, PEG400, and DMSO (1:4:5 v/v, respectively) at a concentration of 1 mg/mL and administered as a single 0.2 mg/kg IV dose (0.5 mL/kg) to male, Sprague Dawley rats (n = 1; 342 gram body weights) via injection into a surgically-implanted jugular vein catheter. Blood samples were collected serially from a surgically implanted carotid artery catheter in each animal over multiple post-administration time points (0.033, 0.117, 0.25, 0.5, 1, 2, 4, 7, and 24 hours) into chilled, K2EDTA anticoagulant-fortified tubes and immediately placed on wet ice. The blood samples were then centrifuged (1700 rcf, 5 minutes, 4 ˚C) in order to obtain plasma samples, which were stored at -80 ˚C until analysis by LC-MS/MS.

For determination of the brain over plasma ratio (K_p), compounds were formulated in 10% ethanol, 40% PEG400 and 50% DMSO (v/v/v) and administered as a single 0.2 mg/kg IV dose (0.5 mL/kg) to male, Sprague Dawley rats (n = 1; 316 gram body weights) via injection into a surgically-implanted jugular vein catheter. At 15 min post dosing, blood sample was collected serially (i.e., terminally) into chilled, K2EDTA anticoagulant-fortified tube and immediately placed on wet ice. The blood sample was then centrifuged (1700 rcf, 5 minutes, 4 ˚C) to obtain plasma sample. At the same post-administration time point, whole brain sample was obtained by rapid dissection, rinsed with saline, and immediately frozen in individual tissue collection box (dry ice). All brain and plasma samples were stored at -80 ˚C until analysis by LC-MS/MS.

Samples preparation for bioanalysis

Plasma samples from the in-life phase of the study were thawed at ambient temperature (benchtop), and then aliquots (20 µL per sample) were transferred to a 96-shallow-well (V-bottom) plate. Matrix-matched quality control (QC) samples and a standard curve of VU6067104 (1 mg/mL DMSO stock solution) were prepared in blank rat plasma (K2EDTA-treated) or blank brain homogenate via serial dilution and transferred (20 µL each) to the plate along with multiple blank plasma and brain homogenate samples. Acetonitrile (120 µL) containing IS (10 nM carbamazepine) was added to each well of the plate to precipitate protein. The plate was then centrifuged (4000 rcf, 5 minutes, ambient temperature), and resulting supernatants (60 µL each) were transferred to a new 96-shallow-well (V-bottom) plate containing an equal volume (60 µL per well) of water (Milli-Q purified). The plate was then sealed in preparation for LC-MS/MS analysis.

Preparation of brain samples was identical to that of plasma samples except for the following modifications. While thawing, brains were weighed (inside their collection boxes using a universal empty collection box tare weight) and then subjected to mechanical homogenization (Mini-BeadBeater™, BioSpec Products, Inc., Bartlesville, OK) in the presence of zirconia/silica beads (1.0 mm) and extraction buffer (isopropanol:water, 7:3, v/v; 3 mL per sample, corrected for post-quantitation). Homogenized brain samples were then centrifuged (4000 rcf, 5 minutes, ambient temperature), and 5 uL of the supernatant was diluted in 15 uL of blank plasma for quantification of the analyte. The plasma standard curve and QCs were used for compounds quantitation in brain.

<u>Binding in plasma from rat and human.</u>

Determination of compounds' fraction unbound (f_u) in plasma from rat and human was conducted *in vitro* via equilibrium dialysis using HTDialysis membrane plates. Dialysis membranes (four paired strips per HTD assay) were hydrated as described by the manufacturer and inserted into the HTD plate, which was assembled and prepared for sample addition by the dispensing of blank buffer (DPBS, 100 µL/well) into the 'top half' of each membrane-split well. Each compound was diluted into plasma from each species (5 µM final concentration), which was aliquoted in triplicate to the 'bottom half' of the prepared HTD plate wells. The HTD plate was sealed and incubated for 6 hours at 37 °C. Following incubation, each well (both top and bottom halves) were transferred (20 µL) to the corresponding wells of a 96-shallow-well (V-bottom) plate. The daughter plates were then matrix-matched (buffer side wells received equal volume of plasma, and plasma side wells received equal volume of buffer), and extraction solution (120 µL; acetonitrile containing 50 nM carbamazepine as IS) was added to all wells of both daughter plates to precipitate protein and extract test article. The plates were then sealed and centrifuged (3500 rcf) for 10 minutes at ambient temperature. Supernatant (60 µL) from each well of the daughter plates was then

transferred to the corresponding wells of new daughter plates (96-shallow-well, V bottom) containing water (Milli-Q, 60 µL/well), and the plates were sealed in preparation for LC-MS/MS analysis (see below).

f_u was calculated as (analyte to IS MS peak area ratio from Trans-buffer side) / (analyte to IS MS peak area ratio from Cis-plasma side). Mean values for each species were calculated from 3 replicates.

Binding in brain homogenate from rat.

Determination of fraction unbound (f_u) in brain homogenate from rat was conducted using the same methodology and procedure than described for plasma protein binding assay with the following modifications: 1) a final compound concentration of 1 µM was used, 2) naïve rat brains were homogenized in DPBS (1:3 composition of brain: DPBS, w/w) using a Mini-Bead Beater™ machine in order to obtain brain homogenate.

The diluted fraction unbound (f_{u2}) in brain was calculated as (analyte to IS MS peak area ratio from Trans-buffer side) / (analyte to IS MS peak area ratio from Cis-brain homogenate side). Undiluted fraction unbound for the brain was calculated using the following equation:

$$f_u = \frac{1/4}{\left\{\left(\frac{1}{f_{u2}}\right) - 1\right\} + 1/4}$$

Mean values for each species were calculated from 3 replicates.

Intrinsic Clearance in Rat and Human Liver Microsomes

The *in vitro* intrinsic clearance (CL_{int}) was investigated in commercially obtained hepatic microsomes from rat and human donors using the substrate depletion (i.e., loss-of-parent vs. time, or $t_{1/2}$ method) approach with analyte detection via liquid chromatography-tandem mass spectrometry (LC-MS/MS). For each species, mean %parent remaining values at each time point were calculated from replicates raw data (analyte:IS peak area ratios) and used to determine *in vitro* $t_{1/2}$ and CL_{int}.

Experiments were carried out using a robot-assisted (TECAN model Evo 200). Compound was incubated (1 µM final concentration) in buffer (100 mM potassium phosphate pH 7.4 with 3 mM $MgCl_2$) containing hepatic microsomes (0.5 mg/mL final concentration) from multiple species, discretely, at 37 °C under constant orbital shaking. After 5 minutes (pre-incubation), reactions were initiated by addition of nicotinamide adenine dinucleotide phosphate (NADPH, 1 mM final concentration). At selected time intervals (0, 3, 7, 15, 25, and 45 minutes) post-addition of NADPH, aliquots (50 µL) were taken and placed into a 96-shallow-well plate containing ice cold acetonitrile (150 µL) with carbamazepine (IS, 50 nM). The plates were then centrifuged (3000 rcf at 4 °C) for 10 minutes. The supernatants were transferred to a new 96-shallow-well daughter plate and diluted (1:1 v/v) with water (Milli-Q filtered). The plates were then sealed in preparation for LC-MS/MS analysis (see below).

Raw LC-MS/MS peak area data generated from the assay samples were used to construct natural log-transformed %parent remaining vs. time plots (using t = 0 minute post-NADPH addition sample data as starting point set to 100%). *In vitro* compound half-life ($t_{1/2}$) values were obtained using the following equation:

$$t_{1/2} = \frac{Ln(2)}{k}$$

Where k is the slope from linear regression analysis of the natural log-transformed data (using means from all replicates at each time point). Resulting $t_{1/2}$ values were then used to calculate hepatic CL_{int} values according to the following equation and with the use of species-specific scale-up factors for liver weight (grams) per total body weight (kg):

$$CL_{int} = \frac{0.693}{in\ vitro\ t_{1/2}}\ x\ \frac{1\ mL\ incubation}{0.5\ mg\ microsomes}\ x\ \frac{45\ mg\ microsomes}{1\ gram\ liver}\ x\ \frac{^{a}\ gram\ liver}{kg\ body\ wt}$$

[a]Scale-up factors used are 45 (rat) and 20 (human).[4]

Predicted hepatic clearance (CL_{hep}) was calculated using the following equation:

$$CL_{hep} = \frac{Q_h * CL_{int}}{Q_h + CL_{int}}$$

Q_h represents hepatic blood flow (mL/min/kg): 21 for human, 70 for rat, and 90 for mouse.

LC-MS/MS Analysis

Prepared samples were injected (10 µL each) onto an AB Sciex Triple Quad 4500 mass spectrometer system with an Agilent 1260 Infinity II pump and autosampler. Mass spectrometer conditions are described in **Table S1.** Quantitation of compounds was performed via AB Sciex Multiquant software using the raw analyte:IS peak area ratios. The typical detection range was 0.5 ng/mL to ≥ 5,000 ng/mL utilizing a quadratic equation regression with 1/x2 weighting.

Correction for dilution of all brain samples (in extraction buffer and subsequently in blank plasma, as previously described) was performed post-quantitation. The corrections for dilution in extraction buffer employed correction factors specific to each brain weight (not shown).

Table S1. **LC-MS/MS Conditions***

Injection volume	10 µL	
Mobile phase A	0.5% Formic Acid in Water	
Mobile phase B	0.5% Formic Acid in Acetonitrile	
Flowrate	0.5 mL/min	
Gradient	Time	% Mobile Phase B
	0.0	5
	0.2	5
	0.8	95
	1.5	95
	1.7	5
	2.7	Stop
Column	Fortis C18 (50 x 3.0 mm, 3 µm)	
Data collection and analysis software/version	Analyst v. 1.7.1	
Ionization mode	Positive Electrospray	
Curtain gas (psi)	40	
GS1 (psi)	40	
GS2 (psi)	40	
Capillary voltage (V)	5500	
Source TurboIonSpray® temp. (°C)	500	

Ancillary Pharmacology

Table S2. Lead Profiling Screen – Eurofins Panlabs for VU6043653.

Assay Name	Species	% inh at 10 μM
Adenosine A_1	hum	20
Adenosine A_{2A}	hum	9
Adenosine A_3	hum	70
Adrenergic α_{1A}	hum	0
Adrenergic α_{1B}	hum	3
Adrenergic α_{1D}	hum	3
Adrenergic α_{2A}	hum	8
Adrenergic β_1	hum	-4
Adrenergic β_2	hum	-5
Androgen (Testosterone)	hum	75
Bradykinin B_1	hum	4
Bradykinin B_2	hum	1
Calcium Channel L-Type, Benzothiazepine	rat	22
Calcium Channel L-Type, Dihydropyridine	rat	1
Calcium Channel N-Type	rat	-8
Cannabinoid CB_1	hum	15
Dopamine D_1	hum	-2
Dopamine D_{2S}	hum	-10
Dopamine D_3	hum	-6
Dopamine $D_{4.4}$	hum	-9
Endothelin ET_A	hum	-2
Endothelin ET_B	hum	-13
Epidermal Growth Factor (EGF)	hum	-3
Estrogen Erα	hum	4
$GABA_A$, Flunitrazepam, Central	rat	0
$GABA_A$, Muscimol, Central	rat	-5
$GABA_{B1A}$	hum	3
Glucocorticoid	hum	10
Glutamate, Kainate	rat	-7
Glutamate, NMDA, Agonism	rat	3
Glutamate, NMDA, Glycine	rat	-18
Glutamate, NMDA, Phencyclidine	rat	-4
Histamine H_1	hum	24
Histamine H_2	hum	-4
Histamine H_3	hum	5
Imidazoline I_2, Central	rat	-1

Interleukin IL-1 R1	hum	-7
Leukotriene, Cysteinyl $CysLT_1$	hum	7
Melatonin MT_1	hum	15
Muscarinic M_1	hum	-13
Muscarinic M_2	hum	-2
Muscarinic M_3	hum	-15
Neuropeptide Y Y_1	hum	-2
Neuropeptide Y Y_2	hum	-5
Nicotinic Acetylcholine α1, Bungarotoxin	hum	-12
Nicotinic Acetylcholine α3β4	hum	6
Opiate δ_1 (OP1, DOP)	hum	3
Opiate κ (OP2, KOP)	hum	2
Opiate μ (OP3, MOP)	hum	9
Phorbol Ester	mouse	-1
Platelet Activating Factor (PAF)	hum	3
Potassium Channel [K_{ATP}]	ham	2
Potassium Channel hERG	hum	14
Prostanoid EP_4	hum	8
Purinergic P2X	rat	15
Purinergic P2Y, Non-Selective	rat	9
Rolipram	rat	-2
Serotonin (5-Hydroxytryptamine) 5-HT_{1A}	hum	3
Serotonin (5-Hydroxytryptamine) 5-HT_{2B}	hum	40
Serotonin (5-Hydroxytryptamine) 5-HT_3	hum	-6
Sigma σ_1	hum	0
Sodium Channel, Site 2	rat	25
Tachykinin NK_1	hum	15
Thyroid Hormone	rat	-2
Transporter, Dopamine (DAT)	hum	25
Transporter, GABA	rat	-1
Transporter, Norepinephrine (NET)	hum	63

Table S3. Tentatively Identified Metabolites of VU6043653 in Rat, Dog, Monkey, and Human Hepatocytes (10 µM incubation for 0 and 4 hours).

Peak ID	Tentative Metabolite Identification	Observed *m/z*	Retention Time (min)	Species/Matrix
VU6043653	Parent (P)	328.1209	7.78	RH, DH, PH, HH
M1	Amide hydrolysis (acid)	234.0871	1.94	RH, DH, PH, HH
M2	P + O + glucuronide	520.1472	4.12	RH, DH, PH, HH
M3	P + O + glucuronide	520.1479	4.17	RH, DH, PH, HH
M4	P + O + glucuronide	520.1493	4.45	DH, PH
M5	P + O	344.1154	4.40	RH, DH, PH, HH
M6	P + glucoside	490.1350	4.58	RH, DH, PH, HH
M7	N-demethylation	314.1051	7.08	RH, DH, PH, HH
M8	P + O	344.1138	5.70	RH, PH, HH
M9	N-demethylation + O	330.0967	4.87	RH, DH, PH, HH
M10	N-demethylation + O + glucuronide	506.1317	4.13	RH, DH, PH, HH

RH – rat hepatocytes, HH – human hepatocytes, DH – dog hepatocytes, PH – monkey hepatocytes

The retention times listed above were obtained from the MS extracted ion chromatograms.

Comments: Ten metabolites were observed in the 4-hour hepatocyte samples. The most abundant metabolite observed with rat, dog, monkey, and human hepatocytes was M1. Overall, the major pathways for VU6043653 appear to be oxidation, demethylation, and glucuronidation.

Proposed Metabolite Structures of VU6043653.

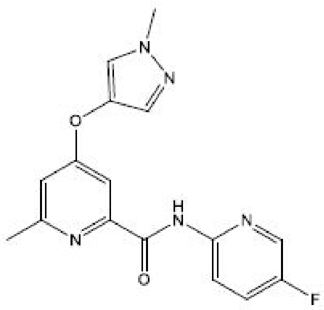

VU6043653, Parent, m/z 328

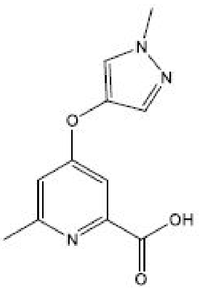

M1, m/z 234

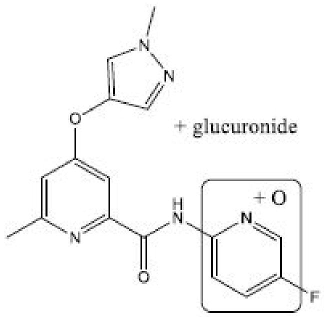

M2, m/z 520

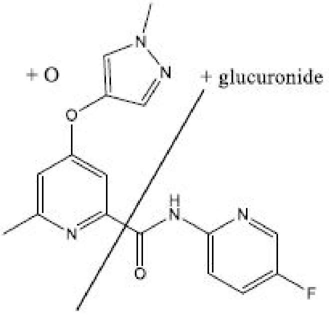

M3, M4, m/z 520

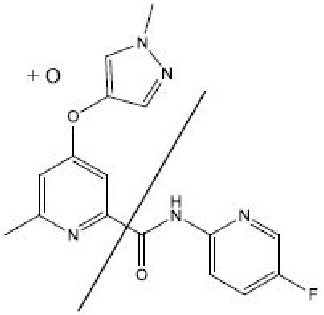

M5, M8, m/z 344

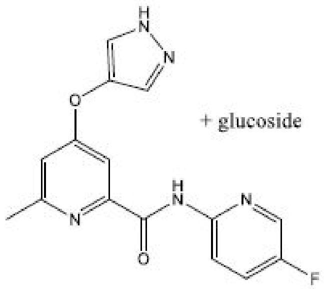

M6, m/z 490

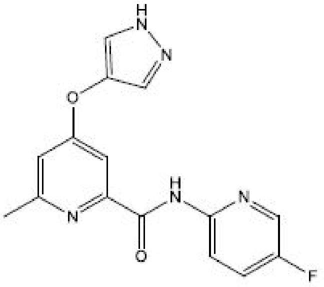

M7, m/z 314

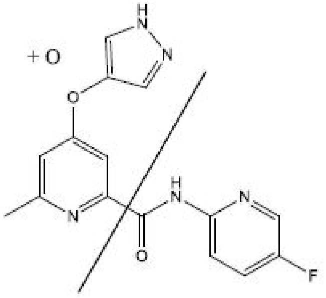

M9, m/z 330

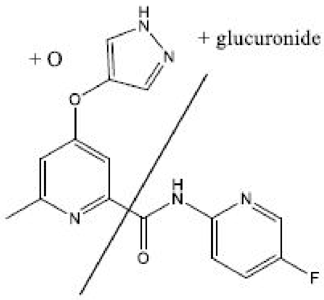

M10, m/z 506

Figure 1. Extracted Ion chromatograms – 4 hr hepatocytes.

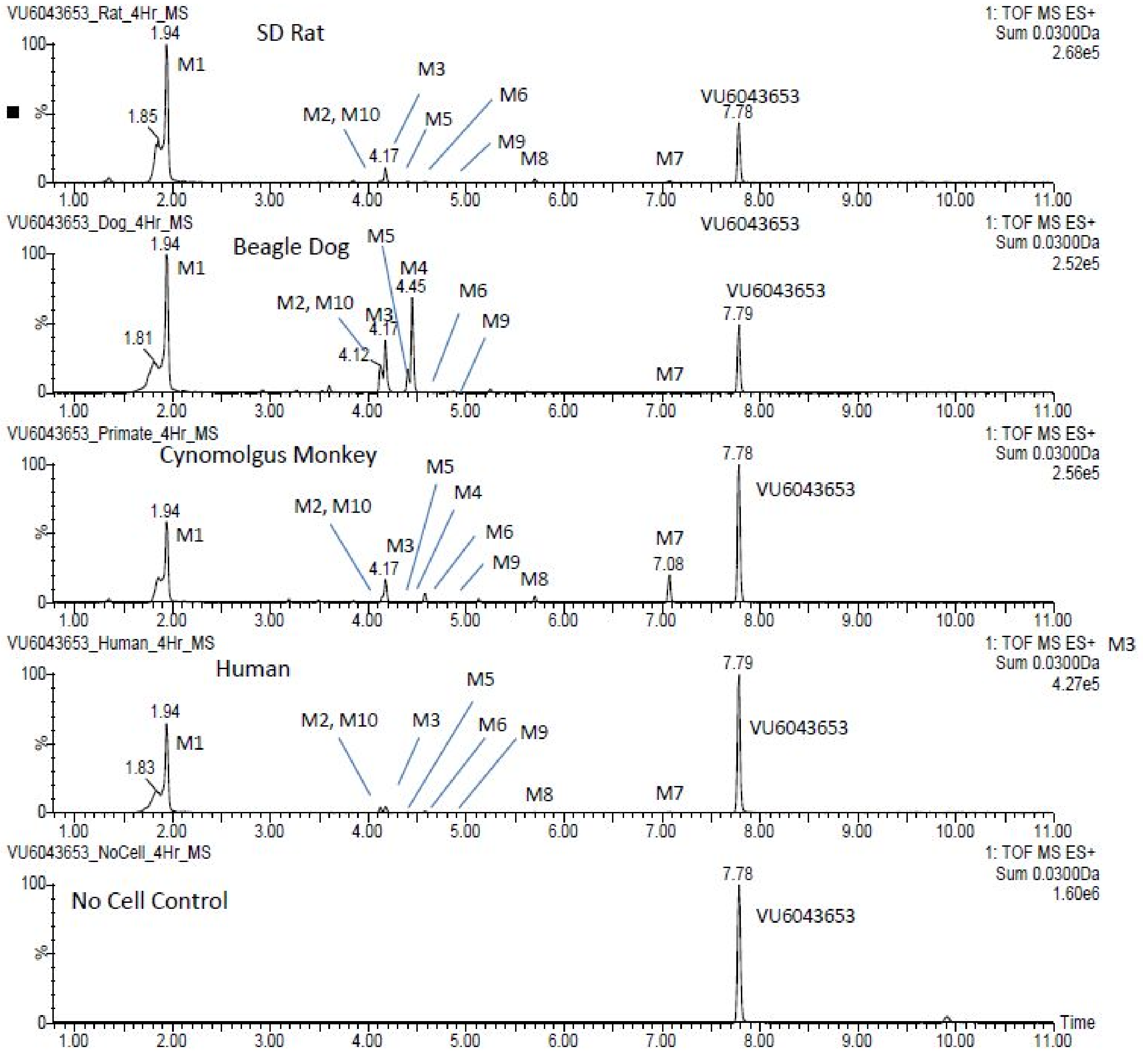

Ions monitored: 328.12+234.09+310.13+344.11+520.15+490.14+314.1+358.09+502.16+330.1+220.07+410.12+344.08+506.13+504.15 +424.07+633.19+326.12+360.11+376.11+447.12+489.14+635.20+449.14+491.15+649.18+463.12+651.20+410.12

	MS Peak Areas										
	Parent	M1	M2	M3	M4	M5	M6	M7	M8	M9	M10
Rat	3926	17225	57	524	ND	75	33	146	246	20	81
Dog	3859	18750	1074	1959	2992	1151	15	12	ND	110	675
Monkey	8424	9624	5	836	15	33	420	1647	380	60	370
Human	14709	19199	330	408	ND	38	122	92	58	1	14
No Cell	59951	ND	ND	ND	ND	ND	ND	ND	ND	ND	ND

References

1) Wu H.; Wang C.; Gregory K.J.; Han G.W.; Cho H.P.; Xia Y.; Niswender C.M.; Katritch V.; Meiler J.; Cherezov V.; Conn P.J.; Stevens R.C. Structure of a class C GPCR metabotropic glutamate receptor 1 bound to an allosteric modulator. *Science*. 2014, 344, 58-64.
2) Reed C.W.; McGowan K.M.; Spearing P.K.; Stansley B.J.; Roenfanz H.F.; Engers D.W.; Rodriguez A.L.; Engelberg E.M.; Luscombe V.B.; Loch M.T.; Remke D.H.; Rook J.M.; Blobaum A.L.; Conn P.J.; Niswender C.M.; Lindsley C.W. VU6010608, a Novel $mGlu_7$ NAM from a Series of *N*-(2-(1*H*-1,2,4-Triazol-1-yl)-5-(trifluoromethoxy)phenyl)benzamides. *ACS Med. Chem. Lett.* 2017, 8, 1326-1330.
3) Niswender C.M.; Johnson K.A.; Luo Q.; Ayala J.E.; Kim C.; Conn P.J.; Weaver C.D. A novel assay of Gi/o-linked G protein-coupled receptor coupling to potassium channels provides new insights into the pharmacology of the group III metabotropic glutamate receptors. *Mol. Pharmacol.* 2008, 73, 1213–1224.
4) Lin J.H.; Chiba M.; Balani S.K; Chen I.W.; Kewi G.Y.; Vastag K.J.; Nishime J.A. Species differences in the pharmacokinetics and metabolism of indinavir, a potent human immunodeficiency virus protease inhibitor. *Drug Metab Dispos*. 1996, 24, 1111-1120.
